# Neuronal circadian clock protein oscillations are similar in behaviourally rhythmic forager honeybees and in arrhythmic nurses

**DOI:** 10.1098/rsob.170047

**Published:** 2017-06-14

**Authors:** T. Fuchikawa, K. Beer, C. Linke-Winnebeck, R. Ben-David, A. Kotowoy, V. W. K. Tsang, G. R. Warman, E. C. Winnebeck, C. Helfrich-Förster, G. Bloch

**Affiliations:** 1 Department of Ecology, Evolution, and Behavior, The A. Silberman Institute of Life Sciences, Hebrew University, Jerusalem 91904, Israel; 2 Neurobiology and Genetics, Biocenter, University of Würzburg, Germany; 3 School of Biological Sciences, University of Auckland, New Zealand; 4 Department of Anaesthesiology, School of Medicine, Faculty of Medical and Health Sciences, University of Auckland, New Zealand

**Keywords:** circadian clock, neuroanatomy, social behaviour, behavioural plasticity, division of labour, *Drosophila*

## Abstract

Internal clocks driving rhythms of about a day (circadian) are ubiquitous in animals, allowing them to anticipate environmental changes. Genetic or environmental disturbances to circadian clocks or the rhythms they produce are commonly associated with illness, compromised performance or reduced survival. Nevertheless, some animals including Arctic mammals, open sea fish and social insects such as honeybees are active around-the-clock with no apparent ill effects. The mechanisms allowing this remarkable natural plasticity are unknown. We generated and validated a new and specific antibody against the clock protein PERIOD of the honeybee *Apis mellifera* (amPER) and used it to characterize the circadian network in the honeybee brain. We found many similarities to *Drosophila melanogaster* and other insects, suggesting common anatomical organization principles in the insect clock that have not been appreciated before. Time course analyses revealed strong daily oscillations in amPER levels in foragers, which show circadian rhythms, and also in nurses that do not, although the latter have attenuated oscillations in brain mRNA clock gene levels. The oscillations in nurses show that activity can be uncoupled from the circadian network and support the hypothesis that a ticking circadian clock is essential even in around-the-clock active animals in a constant physical environment.

## 1. Introduction

Circadian rhythms of about 24 h are ubiquitous in the metazoa and in some bacteria. It is thought that the clocks that generate these rhythms confer an adaptive benefit because they enable organisms to anticipate predictable day–night changes in their environment and align their physiology with these environmental cycles [1]. Studies with humans and model organisms reinforce this notion by showing that disturbing normal circadian rhythmicity by aberrant light–dark illumination or feeding regimes, or by disturbing clock anatomy or clock protein function, increases the risk of many diseases including cancer, metabolic disorders, mental disorders, heart attacks and infertility (reviewed in [2,3]).

Circadian activity rhythms are generated by an endogenous system composed of a network of pacemaker cells capable of autonomously generating rhythms. The molecular mechanism and many of the genes involved in rhythm generation are similar in animals as diverse as fruit flies and mice [4,5]. Input pathways (photic and non-photic) transmit environmental signals to the central pacemaker and keep it synchronized with ecologically relevant day–night cycles. Output pathways carry temporal signals away from the central oscillator to diverse biochemical, physiological and behavioural processes. There is, however, some variability between animals that may use different clock genes in the molecular feedback loops that generate circadian rhythms in pacemaker cells [6]. The honeybee was one of the first animal models in chronobiology (reviewed in [7]). Honeybees allow the study of key chronobiological questions within a clear ecological context. For example, honeybee foragers rely on their circadian clock for timing visits to flowers and for time-compensated sun-compass orientation. Their circadian clock is also involved in the regulation of complex social behaviours such as dance communication and division of labour (reviewed in [8]). Surprisingly, the clock genes encoded by the honeybee genome, as well as their brain transcript expression, are more similar to the mouse than to *Drosophila*. The positive elements in the honeybee rhythm generator are the transcription factors *Clock* (*Clk*) and *Cycle* (*Cyc*), and the negative elements are *Period* (*Per*) and the mammalian-type *Cryptochrome* (*Cry-m*, also termed *insect Cry2*). The honeybee genome does not encode *Timeless1* and the *Drosophila*-type *Cry* which are essential for circadian rhythms in *Drosophila* [6,8,9].

In order to better understand the interplay between circadian rhythms and complex behaviour in honeybees, we developed a new antibody specific towards the clock protein PERIOD (PER) of the honeybee (amPER). We then used this antibody to describe the neuroanatomy of the brain circadian clock network in the bee and in western blot (WB) analyses of whole brain amPER levels. Finally, we sampled bees over a full daily cycle and show that brain oscillations in amPER immunoreactivity in key neuronal clusters are overall similar in foragers with and nurses without overt circadian rhythms.

We chose to focus on plasticity in circadian rhythms because a growing number of reports indicate that some animals naturally show prolonged periods of around-the-clock activity (i.e. comparable levels of activity during day and night) with no apparent ill-effect (reviewed in [10]) or rhythms that are outside the circadian range [11]. Around-the-clock activity with attenuated or no circadian rhythms is not limited to specific phylogenic branches, but rather is associated with certain life-history traits such as seasonal migration in birds or sociality in insects, or specific habitats such as the high Arctic or open sea. The mechanisms underlying natural plasticity in circadian rhythms are yet unknown, and the honeybee provides an excellent model system for addressing this question. Both worker and queen honeybees (*Apis mellifera*) show relatively prolonged periods in their life with no or attenuated behavioural circadian rhythms [8,10]. Plasticity in circadian rhythms in honeybees (and other social insects) is associated with the division of labour that organizes their societies and is therefore functionally significant. Foragers rely on the circadian clock for timing visits to flowers, for time-compensated sun-compass navigation and for sun-guided waggle dance communication. ‘Nurse’ bees, on the other hand, tend brood around-the-clock, which is thought to allow improved care [8,12].

A possible explanation for around-the-clock activity with attenuated or no circadian rhythms states that circadian organization of behaviour and physiology is not crucial or even beneficial for these animals, and their clocks stop when they are active around-the-clock [13]. Alternatively, essential processes that require circadian regulation are under clock control even in animals that are active around-the-clock with no apparent circadian rhythms, but these internal rhythms are masked or uncoupled from the mechanisms controlling overt activity and behaviour. Finally, the mechanism may be more complex such that some processes (such as locomotor activity) are under clock control whereas others are not, or that the phase relationship between internal pacemakers changes under different conditions.

Task-related chronobiological plasticity in honeybees is associated with variation in clock gene expression patterns. Whole brain RNA measurements using various methods show no, or severely attenuated, oscillations in clock gene mRNA levels of around-the-clock active nurse bees relative to foragers, which is consistent with the hypothesis that their clock stops or is not synchronized. Other evidence, however, suggests that at least some pacemakers in their brains do continue to be synchronized with the environment. First, nurse bees that are isolated from the hive rapidly switch to activity with circadian rhythms with a phase entrained to the colony phase [14–16]. Second, microarray analyses suggest that the abundance of about 160 transcripts oscillate in the brain of nurses (compared to about 540 in rhythmic foragers) in cycles with about 24 h rhythm, which is consistent with circadian influence on at least some processes in their brains [17].

Our findings provide the best description of the circadian network in the honeybee brain and set the stage for studies on the interplay between circadian clocks and complex behaviour. Our findings are also important for comparative studies on the neuroanatomical organization of the circadian network in insects. Finally, we provide the first analyses of the circadian network in an animal showing naturally occurring plasticity in circadian rhythms. Our results raise important questions concerning the relationships between oscillations in behaviour and in clock gene expression.

## 2. Material and methods

### 2.1. Cloning and recombinant expression of amPER

We designed cloning primers for full-length amPER based on NCBI GenBank entry NM_001011596.1. The gene for amPER was PCR-amplified from *A. mellifera* forager brain cDNA and cloned into the vector pFastBac (Invitrogen, Carlsbad, CA), which confers an N-terminal His_6_-tag followed by a tobacco etch virus (TEV) protease recognition site on the expressed protein. The sequence of this cloned amPER differs slightly from the above entry and corresponds to the β-isoform of PER as found in the Japanese honeybee [18]. We deposited this sequence in GenBank under accession number KX169182.

### 2.2. Purification of His_6_-amPER

We expressed amPER in insect cell culture and purified it in four steps using immobilized metal-affinity chromatography (IMAC), ion exchange chromatography (IEC) and size exclusion chromatography (SEC). The MultiBac system [19] was used for expression of His_6_-amPer in *Spodoptera frugiperda* Sf9 cells in Sf900 III serum-free medium (Gibco, Gaithersburg, MD). Cells were lysed using sonication in IMAC buffer (50 mM HEPES–NaOH, pH 7.5, 300 mM NaCl, 10% (v/v) glycerol, 20 mM imidazole, 5 mM β-mercaptoethanol, 0.3 mM NaN_3_) + Complete Protease Inhibitor EDTA-free Mini tablets (Roche, Mannheim, Germany) and 100 µg DNaseI (Roche). The lysate was cleared by centrifugation (55 000*g*, 45 min, 4°C), filtered (0.2 µm) and loaded on a HiTrap Chelating HP (GE Healthcare, Chicago, IL) charged with NiCl_2_. We washed the bound protein using IMAC buffer and eluted it using a gradient over 10 column volumes (CV) to a final imidazole concentration of 500 mM. The His_6_-tag was removed using TEV protease (expressed and purified according to Blommel & Fox [20]) under concomitant dialysis against imidazole-free buffer. Undigested protein was removed by a second metal-affinity step. The amPER protein was then loaded on a MonoQ HR 5/5 column (GE Healthcare) using IEC buffer (25 mM Tris–HCl, pH 8.0, 50 mM NaCl, 10% (v/v) glycerol, 2 mM β-mercaptoethanol, 0.3 mM NaN_3_) and eluted using a gradient over 10 CV to a final NaCl concentration of 1 M. Finally, amPER was purified to near homogeneity using a Superdex 200 10/300 column (GE Healthcare) and SEC buffer (20 mM HEPES–NaOH, pH 7.5, 300 mM NaCl, 10% (v/v) glycerol, 2 mM Tris(2-carboxyethyl)phosphine, 0.3 mM NaN_3_). The identity of the purified protein as amPER was confirmed using LC–MS/MS fingerprinting by the Centre for Genomics and Proteomics at the University of Auckland.

### 2.3. Generation and affinity purification of anti-amPER polyclonal antibodies

Polyclonal antibodies were raised against highly purified amPER protein in its native folded state. A mixture of both amPER size species (full-length and truncated protein) was injected into white rabbits by AgResearch (Hamilton, New Zealand) according to standard protocols. Polyclonal amPER-specific antibodies were affinity-purified from serum using purified amPER protein immobilized on CnBr-activated sepharose 4B (GE Healthcare). Bound antibodies were eluted using 100 mM glycine–HCl, pH 2.5, into 1 M Tris–HCl, pH 8.0, dialysed against phosphate-buffered saline (PBS), pH 7.4, and stored with 3 mM NaN_3_.

### 2.4. Immunoprecipitation of amPER

For immunoprecipitation (IP) of amPER, *A. mellifera* foragers were collected in the evening under natural light-dark cycles, transferred to an incubator, and snap-frozen at a time approximately corresponding to zeitgeber time (ZT) 22 (ZT 0 = sunrise), a time during which our preliminary staining experiments indicated that amPER levels are high (which is also consistent with Bloch *et al*. [21]). Complete heads were ground in liquid N_2_ using a mortar and pestle. For each IP, 100 mg of ground heads were thawed and resuspended in 1 ml of IP buffer (50 mM HEPES–NaOH, pH 7.5, 300 mM NaCl) in the presence of protease inhibitors (SigmaFast Protease Inhibitor Cocktail Tablets, EDTA-Free; Sigma, St. Louis, MO). We cleared the lysate using two centrifugation steps (22 000*g*, 10 min, 4°C) and incubated the supernatant with 1 µg of affinity-purified polyclonal rabbit anti-PER antibodies for 1 h at 10°C. We then incubated the lysate with 20 µl nProtein A Sepharose 4 Fast Flow beads (GE Healthcare) for 1 h at 10°C. We washed the beads three times using IP buffer and boiled them in Laemmli buffer. We separated the samples in duplicate lanes using SDS-PAGE for later WB and mass spectrometry analyses. WB was performed as described for the time course analysis (see below), and resulting bands were used to locate bands to be analysed using LC–MS/MS in the duplicate lanes of the SDS-PAGE gel.

For LC–MS/MS analysis, we reduced proteins using 10 mM dithiothreitol, alkylated them using 55 mM iodoacetamide and digested them using trypsin (sequencing grade, Promega, Fitchburg, WI). Resulting peptides were acidified using 1% trifluoroacetic acid, purified by solid-phase extraction using C18 cartridges (Sep-Pak, Waters, Milford, MA) and injected via an EASY-nLC 1000 UHPLC into an LTQ Orbitrap Elite (Thermo Scientific, Waltham, MA) LC–MS/MS system. Peptides were identified using MaxQuant [22], searching against the complete proteome of *A. mellifera*.

### 2.5. Honeybees

Honeybees studied in our experiments were from a mixture of European subspecies typical to Israel and Germany. The bee colonies in Israel were kept at the Bee Research Facility at the Edmond J. Safra campus of the Hebrew University of Jerusalem, Givat-Ram, Jerusalem; honeybees in Germany were kept at the University of Würzburg (Department of Animal Ecology and Tropical Biology). In both facilities, we used standard beekeeping procedures.

For immunocytochemistry studies in Israel, we collected honeybees from triple-cohort colonies. Each colony was placed in an observation hive with transparent walls [16,23,24]. Each colony included a queen and three cohorts of workers: 1500–1800 foragers, 1500–1800 nurses and approximately 1800 newly-emerged (0–24 h of age) worker bees collected from the same source colony. The observation hive contained two honeycomb frames: the upper frame was empty for the queen to lay eggs, and the lower frame was filled with pollen and honey. We placed the observation hive in an environmental chamber (approximately 30 ± 1°C, relative humidity (RH) 45 ± 10%) maintained under dim red light illumination (DD; illuminated with Edison Federal EFEE 1AE1 Deep Red LED light; mean wavelength = 660 nm, minimum = 650, maximum = 670) except during the light phase of a light–dark (LD) illumination regime (see below). The observation hive was connected to the outside with a 1.5 m ‘S’-shaped plastic tube (diameter 3 cm) covered with aluminium foil. The shaping and aluminium foil cover of the entrance tube allowed the bees to exit the hive and freely forage for food but prevented direct exposure of the inner parts of the observation hive to sunlight. We placed a gate made of a sliding plastic sheet next to the tube edge next to the hive entrance (inside the environmental chamber). This manually controlled gate allowed us to control the access of bees to the outside [15].

The bees for WB analyses and immunocytochemistry (co-staining for amPER and anti-pigment-dispersing hormone (PDH) and/or horseradish peroxidase (HRP), and 4′,6-diamidino-2-phenylindole dihydrochloride hydrate (DAPI)) in Germany were collected from field colonies housed in three-story Langstroth standard hives. These colonies consisted of approximately 35 000–40 000 bees; the two lower supers contained the brood nest, which spanned over six to seven frames; a queen excluder was placed between the two upper supers. For the WB samples of nurse bees, we introduced (approximately 120) newly emerged worker bees into a two-frame observation hive (established a few months before) with a queen and approximately 4000 worker bees. All bees in the WB experiments were sisters from the same source colony headed by a naturally mated queen. Unlike the studies in Israel, this observation hive was housed outside, and not in an environmental chamber and, therefore, could be less well thermoregulated (due to logistic constraints).

### 2.6. Collection of forager bees at two time points for immunocytochemistry; Germany

Foragers of unknown age were collected with a pooter from flowers close to the hive or returning to the hive after foraging. Half of the bees were dissected immediately (ZT 11), and the other half were placed in a cage in an incubator with an LD illumination regime (20°C, approximately 200 lux during light phase; approx. 10–15 bees/cage, supplied with sugar syrup) fitting with ambient dawn and dusk and collected the following night (ZT 22). The ambient LD regime varied between experiments due to strong variation in daylight time during the year in Germany and was approximately 11 : 13 in the wholemounts experiment (lights off = 18.00) and approximately 16.5 : 7.5 for the vibratome experiments (lights off = 21.30). Time course experiments were not performed in Germany.

### 2.7. Collection of nurse and forager bees around-the-clock for immunocytochemistry; Israel

We first conducted a preliminary experiment (2–3 October 2013) with bees from a different source colony than those used in the main experiment. In this experiment, we collected (10.00–11.00) only foragers returning to the hive (identified as below) and divided them into five groups of 15 bees each. Each group was placed in a separate wooden cage (11 × 10 × 4.5 cm with Plexiglas cover), provisioned with 4–5 ml of 50% (w/v) sucrose syrup. All the cages were housed in the same environmental chamber (28 ± 1°C, 55 ± 10% RH, fluorescent light-on until 19.00, and then switched to constant darkness). We started to collect samples for immunostaining at 22.00 (circadian time (CT) 15.00) of the same day and continued sampling bees every 6 h (in DD) until 22.00 on the second day (five time points spanning over an entire day). Other details were similar to the main experiment (see below).

In the main experiment, we established a triple-cohort colony in an observation hive as described above. We paint marked (Humbrol enamel paint, UK) 200–400 newly emerged bees and approximately 200 foragers with different colours (i.e. white for newly emerged bees, gold for foragers). During the first two days, we kept the observation hive in DD and the hive entrance closed to allow the bees to adjust to the observation hive. During days 3–7, we maintained the hive in a 12 h L : 12 h D illumination regime (light-on 6.00, 8.00; light-off 18.00, 20.00, for trials 1 and 2, respectively). The observation hive was illuminated with fluorescent lamps emitting light (200 lux) that is potent enough to entrain honeybees [15,16,25]. We opened the hive entrance when the lights were turned on and closed it when the lights were turned off such that the foragers and nest bees experienced similar light and dark phases. During the two days before collecting the bees for immunostaining (days 6 and 7, see electronic supplementary material, figure S1), we observed foraging behaviour in front of the hive and paint-marked foragers (approx. 300) that could be collected later in DD. During the early night of day 7, we changed the illumination to dim red light, detached the entrance tube and connected it to a foraging arena that was placed inside the constantly dark (DD) environmental chamber. We provisioned the foraging arena with a Petri dish (diameter 150 mm) filled with 50% (w/v) sucrose syrup. We sampled foragers and nurses (5–10 bees for each group/time point) for PER immunocytochemistry starting at 22.00 on day 7, and every 6 h until the last (fifth) collection at 22.00 on day 8. Thus, the same time point (22.00) was sampled twice on two successive days. We identified nurses as 7-day-old bees (known based on the colour of their paint-mark) observed with their head inside a honeycomb cell with an egg or a larva. We identified foragers by the gold paint dot on their thorax. The bees were immediately chilled on ice and kept cold until their brains were dissected (the entire procedure from collection to fixation took 1–2 h). We repeated this experiment twice (on 16–24 March 2014 and 16–23 June 2014), with bees from two different source colonies (colonies 13-13 and 13-20, respectively).

### 2.8. Western blot time course analysis, Germany

Newly emerged bees were paint marked and reintroduced into their hive. Nurse bees and foragers were collected after 7 and 24 days, respectively. Nurse bees were collected from the brood area, many of which were also seen tending brood cells. Nurse-age and forager bees selected based on their age were collected from the hive on the evening before the time course sampling and transferred into Plexiglas cages (12 cm^2^ × 8 cm) with about 30 similar bees. The cages were then placed in a climate chamber (20°C, switching to DD at the time of natural sunset. Forager and nurse-age experiment: lights off 20.00 CEST, LD approximately 13.5 : 10.5; nurse experiment: ambient LD regime approximately 15 : 9, lights off at 21.00) until sampling. The bees experienced a short light exposure (5–10 min) when the hive was opened for collecting the nurse bees. We collected samples of bees every 4 h starting 4.5 h after lights off. The bees were flash frozen in liquid nitrogen and stored at −80°C until further analyses. We dissected the brains on dry ice and kept the tips of the forceps chilled in liquid nitrogen to ensure brains stayed frozen during the entire dissection procedure.

For the WB time course experiments, we pooled five bee brains per time point. The brains were suspended in 100 µl lysis buffer (125 mM Tris–HCl, pH 6.4, 10% v/v glycerol, 4% w/v sodium dodecyl sulfate (SDS), 4 M urea, 0.001% w/v bromophenol blue, 1% (v/v) β-mercaptoethanol) supplemented with protease inhibitors (SigmaFast Protease Inhibitor Cocktail Tablets, EDTA-Free, Sigma, St. Louis, MO) and disrupted using a rotor stator homogenizer or a bead beater. The lysate was cleared using centrifugation (20 000*g*, 15 min, 4°C) and the supernatant was boiled. Samples were separated using SDS-PAGE (NuPAGE Novex 4–12% Bis-Tris gels, Life Technologies, Carlsbad, CA) and blotted on nitrocellulose or polyvinylidene difluoride (PVDF). The membrane was blocked using 5% (w/v) milk in TBST (Tris-buffered saline, pH 7.4, 0.1% (v/v) Tween-20) and cut into an upper and a lower half. The upper half was incubated with the affinity-purified anti-amPER polyclonal antibodies (0.25 µg ml^−1^), the lower half with mouse anti-β-actin C4 (Santa Cruz Biotechnology, Santa Cruz, CA, diluted 1 : 2000) overnight at 4°C. All antibodies were diluted in 1% (w/v) milk in TBST. After washing, membranes were incubated with anti-rabbit-HRP (Bio-Rad, Hercules, CA, diluted 1 : 1000; or GE Healthcare, diluted 1 : 10 000) for amPER or with anti-mouse IgG1-HRP (gift of Elisabeth Kremmer, Helmholtz-Zentrum Munich, diluted 1 : 1000) for β-actin. After washing, blots were developed using SuperSignal West Femto Maximum Sensitivity Substrate (Life Technologies) or a self-made ECL substrate (100 mM Tris–HCl, pH 8.5, 0.03% H_2_O_2_, 225 µM cumaric acid, 1.25 mM luminol) and visualized using film.

For quantification, films were scanned (transmission mode) and those with the lowest still informative exposure times selected and checked for saturated stains, before bands of interest were measured densitometrically using ImageJ (National Institutes of Health, Bethesda, MA; version 1.48, according to the ImageJ user guide). We calculated the relative band intensity by dividing the relative density of the band of interest by the relative density of the respective loading control band (β-actin) and normalized it relative to the time point with the highest density.

### 2.9. Immunocytochemistry using the new anti-amPER antibody

We performed PER immunocytochemistry on wholemount brains or vibratome sections.

#### 2.9.1. Vibratome sections

We chilled the collected bees on ice, and when gathering all the samples for a given time point, transferred all the sampled bees on ice from the Bee Research Facility to the laboratory. We then promptly opened the head capsule cuticle and immersed the tissue in a fixation solution (4% paraformaldehyde (PFA) in PBS with 0.1% Triton). We separated the head from the thorax and immersed the head in fixation solution for an additional 3 h (±15 min) at room temperature. At the end of fixation, we transferred the heads to PBS (pH 7.4, Sigma, P3813; St. Louis, MO) solution and completed dissecting the head capsule. We gently removed the brain and embedded it in 4% Bacto agar (BD Difco 214 010; Sparks, MD, USA), in which it was kept at 4°C until sectioning (less than 1.5 months). We used a Leica VT1000 vibratome to slice the embedded brains into 80 µm frontal sections. We washed (2 × 5 min) the sections in PBS and incubated them for 1 h at room temperature in a blocking solution containing 10% normal goat serum (NGS; Fisher Scientific 31873; Rockford, IL) in PBST (PBS with 0.5% Triton X-100). At the end of blocking, we drained the blocking solution and incubated the tissue with the new rabbit anti-AmPER antibody. We diluted the antibody 1 : 1000 in a solution containing 5% NGS and 0.02% NaN_3_ in PBST (thereafter referred to as ‘diluting solution’). We chose this dilution based on a set of preliminary experiments in which we tested paraffin and vibratome sections and dilutions ranging from 1 : 3000 to 1 : 100 (data not shown). We incubated the tissue with the primary antibody in a humidified chamber overnight at room temperature. After incubation with the primary antibody, we washed the sections for 6 × 20 min in PBST and incubated them overnight with a secondary goat anti-rabbit antibody (Alexa Fluor 488; Invitrogen A11008; Eugene, OR; diluted to 1 : 500 in diluting solution) at room temperature. We washed the sections again (6 × 20 min) in PBST. In the fifth wash, we mixed the washing solution with 1 mg ml^−1^ DAPI (Sigma D9542; Eugene, OR) and mixed thoroughly (1 : 400 dilution) to allow for a nuclear counterstaining.

#### 2.9.2. Wholemount brains

The sampling and the fixation of the bees processed as wholemounts were very similar, except for the fact that the incubation time for the primary antibody was increased to 7 days (6 days at 4°C and 1 day at room temperature) and the incubation times for the secondary antibodies to 1 day. Furthermore, we added an antigen retrieval step by incubating the brains in a sodium citrate buffer (10 mM, pH 8.5) for 20 min in an 80°C water bath before starting immunostaining. We thoroughly washed off the sodium citrate buffer with PBS before the blocking step. The mounting of the brains was conducted with the help of spacers which prevented the slides from crushing the tissue.

### 2.10. Wholemount immunocytochemistry for *Drosophila melanogaster* brains

To test the specificity of the new anti-amPER antibody, we collected Canton-S and *per*^*01*^ mutant *D. melanogaster* flies at ZT 22 (when dmPER levels are expected to be high [26]) and fixed the entire fly for 3 h in 4% PFA (in PBST with 0.1% Triton) at room temperature. We dissected the brains in PBS under a binocular microscope. We then separated the head and opened the head capsule, and after removing all the surrounding tissue, removed the brain and washed it three times in PBS and three times in PBST (0.5% Triton) (10 min each). After blocking in 5% NGS–PBST (0.5% Triton) overnight at 4°C, we applied the primary anti-amPER antibody (dilution: 1 : 1000; in diluting solution) and incubated it for 3 days at room temperature. At the end of incubation, we washed the tissue six times (10 min each) in PBST (0.5% Triton) and incubated it with a secondary antibody (diluted 1 : 400 in 5% NGS–PBST (0.5% Triton); goat anti-rabbit IgG (H + L) secondary antibody, Alexa Fluor 488, Catalog#: A-11008, ThermoFisher Scientific, Waltham MA, USA) solution overnight at 4°C. Following incubation, we rinsed the tissue six times in PBST (0.5% Triton). For PER–pigment-dispersing factor (PDF) double labelling immunostaining, we fixed the brains overnight at 4°C in Zamboni's fixative (4% PFA, 7.5% saturated picric acid solution in 0.1 M PBS, pH 7.4), which is optimal for immunostainings against neuropeptides. After incubating the brains with the first secondary antibody, we fixed the brains and washed them 10 times in PBS (10 min each), and then immunostained with an antibody against PDH. We used an antibody that was raised against the *Uca pugilator* PDH (1 : 3000 [27]; provided by Heinrich Dircksen, Stockholm University, Sweden), which is the crustacean homologue of PDF. This rabbit anti-PDH antibody recognizes insect PDF peptides, including *Drosophila melanogaster* [28] and *A. mellifera* PDF [21,29], and thus allowed us to keep similar immunostaining protocols for the fly and the bee. The anti-PDF antibody was applied for 4 days at 4°C and 1 day at room temperature. After washing (as above), we applied an Alexa 635-conjugated goat anti-rabbit secondary antibody (diluted 1 : 400 in 5% NGS–PBST (0.5% Triton), goat anti-rabbit IgG (H + L) secondary antibody, Alexa Fluor 635, Catalog no. A-31576, ThermoFisher Scientific). Brains were washed three times in PBST (0.5% Triton) and three times in PBS (10 min each) and mounted in Vectashield (Catalog no. H-1000; Vector Laboratories, Burlingame, CA). The complicated procedure was necessary because the amPER and PDF antibodies were both raised in rabbits. The second round of fixation denaturized the amPER antibody, so that it could no longer be detected by the fluorescent secondary antibody used to visualize PDF.

### 2.11. Double-labelling brain tissue with anti-amPER and anti-PDF

#### 2.11.1. Vibratome sections (Israel)

At the end of incubation with the secondary antibody, we thoroughly washed the sections (6 × 20 min) in PBST and incubated them in blocking solution for 1 h at room temperature. After draining the blocking solution, we incubated the tissue with the rabbit anti-PDF antibody (raised against the PDF peptide of the cricket *Glyllus bimaculatus* [30]; courtesy of Dr Kenji Tomioka, Okayama University, Japan), diluted 1 : 1000 in diluting solution, overnight at room temperature. Following incubation, we washed the tissue in PBST (6 × 20 min) and incubated it for 8 h with a goat anti-rabbit Alexa Fluor 546-conjugated secondary antibody (Invitrogen A-11010 diluted to 1 : 500). Given that both the anti-amPER and the anti-PDF antibodies were generated in rabbit, the immunostaining should be interpreted in the following way. (i) Cellular structures labelled with both Alexa Fluor 488 and Alexa Fluor 546 fluorophores may express either both PER and PDF, or only PER. This latter possibility may occur if not all available sites on the anti-amPER antibodies were bound by the secondary goat anti-rabbit Alexa Fluor 488-conjugated secondary antibody, and therefore some were also labelled by the later added Alexa Fluor 546-conjugated secondary antibody. We believe that this is not very likely given the high concentrations and long incubation time with the secondary antibody following the incubation with the anti-amPER antibody. (ii) Cellular structures labelled only with Alexa Fluor 488 fluorophore express PER but not PDF. (iii) Cellular structures labelled only with Alexa Fluor 546 fluorophore express PDF but not PER.

#### 2.11.2. Wholemount brains (Germany)

Here we used the anti-PDH antibody (diluted 1 : 300) for double-staining the amPER-immunostained brains. We fixed the brains for a second time in Zamboni's fixative before starting anti-PDH labelling (see §2.10 above). As secondary antibody we used goat anti-rabbit Alexa Fluor 635-conjugated antibody (dilution 1 : 300, applied for one day). By this method, we obtained amPER stained in green and PDF stained in infrared without any overlap of staining.

### 2.12. Triple staining with amPER, horseradish peroxidase and DAPI

We performed triple labelling to assess the neuronal/glial nature of the PER-immunoreactive cells, as well as the sub-cellular location of PER. We performed a similar procedure to that detailed above for anti-amPER immunostaining on vibratome sections. To reduce background staining, we pre-absorbed the amPER antibody on embryos of *per^01^ D. melanogaster* mutants. The embryos were dechorionated in bleach, fixed in formaldehyde/heptan mixture (1 : 1) and devitellinized in methanol. After a stepwise rehydration in 50% methanol/PBST (0.5% Triton) solution to PBST (0.5% Triton), the antibody was incubated in a 1 : 100 PBST (0.5% Triton)–NGS (5%)–NaN_3_ (0.02%) dilution for 1 h at room temperature. The pre-absorbed anti-amPER antibody was used at a 1 : 100 dilution. For the HRP staining, we incubated the tissue with an anti-HRP antibody (Cy3-AffiniPure Goat Anti-Horseradish Peroxidase, Catalog no. 123-165-021; Jackson ImmunoResearch, West Grove, PA). After overnight blocking at 4°C, brains were incubated in the antibody solution (1 : 300 PBST (0.5% Triton)–NGS(5%)–NaN_3_ (0.02%)) 48 h at room temperature and washed five times in PBST (0.5% Triton). In the fifth washing step, we added DAPI for the nuclear counterstaining (procedure stated above) and washed three times in PBS.

### 2.13. Assessing the intensity of PER immunoreactivity

In Jerusalem, we used a laser scanning confocal microscope (TCS-SP8, Leica) equipped with 40×/1.30 oil HC PL APO CS2 objective to excite the fluorophores and photograph the tissue (at a scan speed of 400 Hz). In Würzburg, we used a Leica confocal microscope (Leica TCS SPE) equipped with a 10×/0.30 CS ACS APO and a 20×/0.60 IMM CORR ACS APO objective. The Alexa Fluor 488 fluorophore (PER-immunoreactive (ir)) was excited at 488 nm, and the emission was detected with wavelength at the range of 496–541 nm. The Alexa Fluor 546 fluorophore (PDF-ir) was excited at 561 nm, and the emission was detected with wavelength in the range of 569–620 nm. The detector gain and laser power for PER-ir signal detection were adjusted to the levels at which saturation does not occur using the image of lateral clusters of neurons (LN_1_, see Results) at 10 h in DD (CT 22) in foragers studied in the first trial, which are the maximal levels (gain: 109 and 200 for trial 1 and 2, respectively, laser power: 3% for both trials (argon laser output was set to 30% (=19.5 mW))). The parameter settings were kept constant within each trial of the nurse/forager experiment (Israel). In the preliminary experiment, we used different parameter settings but, again, kept them constant throughout the trial. We photographed stacked images of three focal regions (dorsolateral cluster, lateral cluster and optic lobe) in both hemispheres in each 80 µm immunostained vibratome section. We typically saw the focal areas in either the most frontal, second or third section. Cell clusters that were separated between two sections were not included in our analyses.

We used the ImageJ v1.45 software to quantify the average fluorescent intensity inside each PER-positive cell. For obtaining peak fluorescence from sequential images, we carefully chose images that showed the highest fluorescent intensity compared with several successive images from a focal cell. We used the DAPI staining to define the nucleus of the focal cell. In all the cells in which we quantified amPER immunostaining intensity, the amPER-ir signal appeared to be limited to the nucleus. We calculated the cluster average based on the intensity measured for individual cells. We normalized the signal intensity in focal cells relative to background signal intensity to account for possible technical variation in the immunostaining of different brains. Background signal intensity was measured in three distinct fields (each comparable in size to a PER-positive cell) in the vicinity (range 2–44.5 µm, mean 12.2 µm) of the PER-positive cells. We normalized staining intensity by subtracting from each focal cell the background signal intensity calculated by averaging the intensity for the three adjacent background fields. For each cluster except for the LN_2_ cluster, we calculated the average normalized signal intensity for the 15 cells with the strongest signal intensity in one brain hemisphere. In tissues in which we could detect less than 15 positive PER-ir cells per cluster (i.e. because signal intensity was low), we recorded the signal intensity for all the PER-ir cells and assigned 0 for the rest of the values needed to reach a sample size of 15 cells. For the LN_2_ cluster, we calculated the average normalized signal intensity for all the cells in the cluster.

To estimate the degree of synchronization among individual amPER-ir neurons within the LN_2_ cluster, we calculated the coefficient of variance (CV) of amPER-ir intensity and compared synchronization for nurses and foragers over the five time points. The CV was calculated as the standard deviation of amPER-ir intensity of individual cells divided by the cluster average. The CV is relatively large when variability among cells in the same cluster is large, which occurs when the oscillations of PER expression are less synchronous among cells.

### 2.14. Statistical analyses

We used two-way ANOVA with time of day and task (nursing or foraging specialization) to compare the normalized intensity of amPER-ir signal in the three PER-ir neuron clusters (DLN, LN_1_ and LN_2_) and glial cells in the optic lobe, as well as for the CV values of the LN_2_ cells. Given the small sample size, we performed complementary Kruskal–Wallis tests followed by Nemenyi post hoc tests [31] separately for the forager and nurse data. Sample size of each time point is four for the preliminary experiment and for the first trial, and six for the second trial. To determine if the temporal pattern of expression fits a cosine model, we performed regression analyses to a cosine model using the CircWave v. 1.4 software [32] with an assumed period of 24 h. We used the JMP software v. 8.0.2 (SAS Institute Inc., Cary, NC) for all statistical analyses besides Nemenyi tests. For the Nemenyi tests, we used the R package PMCMR v. 4.1 [33].

## 3. Results

### 3.1. Characterization of a newly generated anti-*Apis mellifera* PERIOD (anti-amPER) antibody

**Figure 1.**
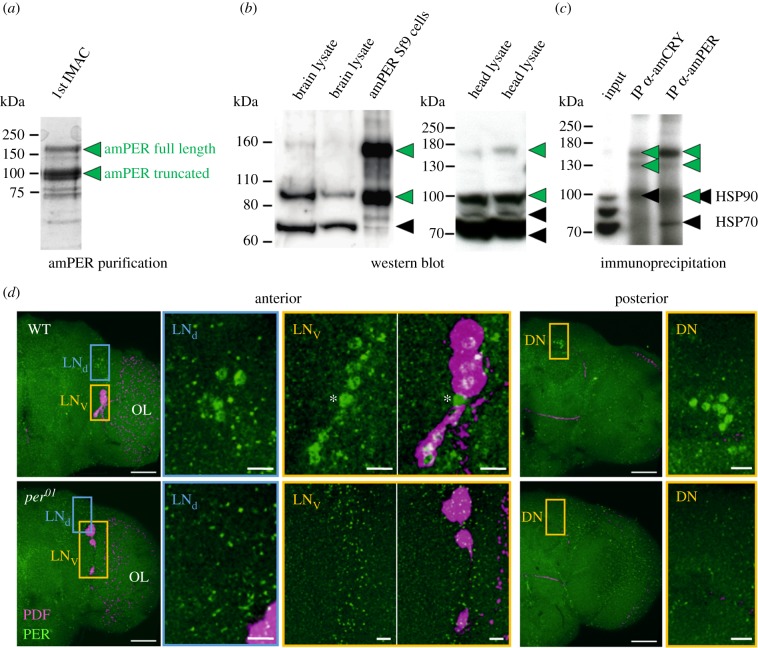
Antibody production and validation. (*a*) Purification of recombinant amPER for rabbit immunization. Two species of amPER were apparent throughout the purification process. Displayed is a single fraction from the first step of amPER purification via IMAC separated on SDS-PAGE (Coomassie staining). (*b*) Validation of antibody in WBs. In bee tissue extracts, the amPER antibody detects up to four main bands (plus occasional laddering of top band), of which two correspond directly to the two species of recombinant amPER observed in Sf9 cells (green arrowheads, see also (*a*)). The other two bands likely result from unspecific binding (black arrowheads). (*c*) Validation of antibody using IP followed by mass spectrometry analysis. The amPER antibody enriches only amPER-specific signals (green arrowheads) in IP from bee head lysates but gives some unspecific signal in WBs (black arrowheads). Displayed is an anti-amPER WB of IPs with either amPER or amCRY antibody. (*d*) Validation of antibody in immunohistochemistry. Double staining with antibodies against amPER (green) and PDF (magenta) in wholemount brains of *D. melanogaster* wild-type (WT) and *period* null mutant (*per^01^*) flies. In the anterior right brain hemisphere of WT flies, the nuclei of the typical LN_d_ and LN_v_ cell clusters are stained with amPER. All cells of the LN_v_ cluster, except one (asterisk), co-express PDF. In the posterior right brain hemisphere, the nuclei of the DNs are labelled. In *per^01^* brains, none of the cell clusters was labelled by amPER, only unspecific staining (green dots) can be seen. OL, optic lobe. Scale bars in hemisphere views: 50 µm. Scale bars in magnified pictures: 10 µm. Pictures are taken with a 25× objective (0.6 numerical aperture); distance of *z*-stacks: 1 µm (six overlaid stacks in the anterior brain and 22 overlaid stacks in the posterior brain).

Antibodies against *A. mellifera* PERIOD protein (amPER, predicted size = 128 kDa) were raised using a highly purified amPER protein in its native folded state recombinantly expressed in insect cell culture from forager brain cDNA. Initial purification of the N-terminally His-tagged amPER protein led to two protein species, a minor species with apparent size in gel electrophoresis of 160 kDa and a major one of about 100 kDa (figure 1*a*). Using mass spectrometry fingerprinting, both species were confirmed as amPER protein, the 160 kDa species being the full-length protein and the 100 kDa species a C-terminally truncated form. After removal of the tag and further purification to homogeneity, amPER (a mixture of both species) was used for rabbit immunization. Polyclonal antibodies against amPER were finally affinity-purified from rabbit serum using purified amPER protein produced the same way as for immunization and including both size species.

The affinity-purified amPER antibody was characterized and validated using WB, IP, mass spectrometry and immunohistochemistry (figure 1) in line with recent suggestions on antibody validation [34]. In WB using lysate from amPER-overexpressing cells, the antibody recognizes exactly two proteins running at the same height as both species of purified amPER (figure 1*b*). In lysates of bee heads and brains, up to four main bands are observed (with the top one showing some laddering in some instances) (figure 1*b*). Bands at 70 and 80 kDa likely result from unspecific binding of the antibody (the 80 kDa band is stronger when using whole heads instead of brains or in cases of glandular contamination in brain preparation). The bands at 160 and 100 kDa correspond directly to the two amPER species observed in recombinant expression, only with lower intensity, indicating target-binding of the antibody. This also means that amPER was found to exist as two species both in lysates from recombinant expression culture as well as from honeybee tissue.

IP of endogenous amPER from whole *A. mellifera* heads followed by WB led to a clear enrichment of the two species at 160 and 100 kDa (figure 1*c*, lane 3). Mass spectrometry analysis of the immunoprecipitated proteins unambiguously identified these proteins as full-length amPER for the 160 kDa band and C-terminally truncated amPER for the 100 kDa band. The antibody thus recognizes amPER both in its folded (IP) and unfolded (WB) states. Our mass spectrometry analysis of the IP also revealed that *A. mellifera* Cryptochrome (amCRY-m) was co-precipitated with amPER. This is in agreement with previous observations in other organisms that the mammalian type CRY-m and PER may form a complex [35]. Interestingly, when we performed IP of amCRY (using an antibody that will be described elsewhere), only the 160 kDa full-length amPER species but not the C-terminally truncated 100 kDa species was co-precipitated (figure 1*c*; the band at 100 kDa in lane 2 contains mainly HSP90 but no amPER according to mass spectrometry). Thus, our results are consistent with the premise that amPER, like other PER homologs, interacts with CRY-m through a C-terminal domain [35–37].

Importantly, our analyses of the IP did not identify enrichment of proteins other than amPER and amCRY-m (figure 1*c*), indicating specificity of the antibody for amPER in solution under native conditions. However, the combination of WB, IP and mass spectrometry analyses identifies some cases of unspecific binding of the antibody in WB: the band at 70 kDa likely represents HSP70, whereas the protein leading to the 80 kDa signal remains unidentified. Furthermore, the specific signal at 100 kDa for truncated amPER might be contaminated with an unspecific signal through antibody binding to HSP90. This notwithstanding, the 160 kDa band observed in WB can be fully attributed to full-length amPER.

In the absence of a *per* knock-out mutant of *A. mellifera*, we turned to *Drosophila melanogaster* for further validation of the new amPER antibody in immunocytochemistry. Applying the anti-amPER antibody to wild-type *D. melanogaster* brains, we detected several weakly stained PER-ir neurons that correspond to known PER-ir clock cell clusters (figure 1*d*) [38]. These clusters were stained in all of 16 analysed wild-type wholemount brains. In contrast, parallel staining of brains from *per^01^* mutant *Drosophila*, which do not express PER, did not reveal these clusters in any of the 10 analysed brains. In both wild-type and *per^01^* mutants, we additionally found small dots of putatively unspecific staining throughout the brain (figure 1*d*). Unspecific signal is easily distinguishable from the specific nuclear staining seen in wild-type brains and seems to be more abundant in the *per^01^* brains. Thus, our amPER antibody is suitable for immunocytochemistry and specifically detects *Drosophila* PER, which has an amino acid sequence similarity to amPER of 55% [39]. Taken together, the above validations show that our amPER antibody is specific for folded amPER, although some unspecific reactions may occur in the context of unfolded proteins as are used in WBs. This finding is in line with our immunization and affinity-purification strategy that used amPER in its native folded state for both steps.

### 3.2. Whole brain amPER levels oscillate in western blot analyses of foragers, nurses and nurse-age bees

**Figure 2.**
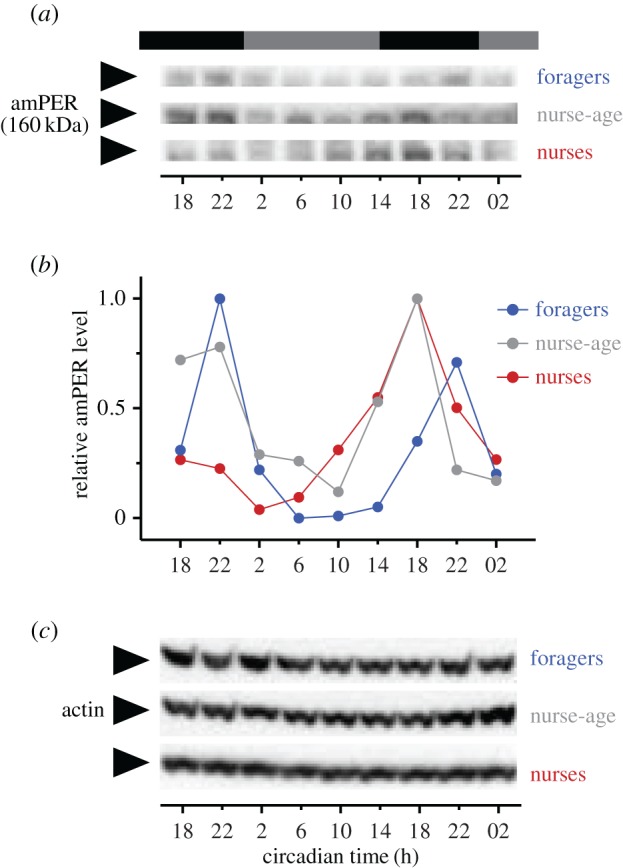
Time course analyses of amPER immunoreactivity in whole brains. Forager, nurse-age and nurse bees were sampled over 1.5 days in constant darkness and amPER brain expression analysed by WBs (*n* = 5 pooled brains per time point, 1 time course each). The band representing full-length amPER (*a*) oscillated in intensity relative to the loading control actin (*c*), the quantification of which is displayed in (*b*). Photoperiod prior to DD is indicated in black and grey bars above the graph. The term ‘nurse-age’ refers to bees of 7–9 days of age that were removed from the brood comb several hours before the onset of sampling.

For a first temporal expression analysis of amPER in bee brains, we performed WBs on whole brain lysates from samples collected over 32 h under constant darkness (DD) and quantified the specific amPER 160 kDa band. amPER protein levels oscillated in a circadian manner in forager brains (figure 2), which is consistent with the oscillating *per* mRNA levels [6,16,24,39–41]. Peak abundances were found at CT 22. Brain amPER levels appear to also oscillate in nurse bees sampled directly from the brood comb as well as nurse-age bees sampled from outside the hive (figure 2). Quantification of the 100 kDa band (truncated amPER + HSP90) suggested similar circadian oscillations in amPER levels in foragers, albeit with less robust peak times, but none in nurse or nurse-age bees (not shown). Given these interesting findings, we embarked on an in-depth analysis of temporal and spatial amPER expression in bee brains.

### 3.3. Many cells in the honeybee brain are amPER-immunoreactive

**Figure 3.**
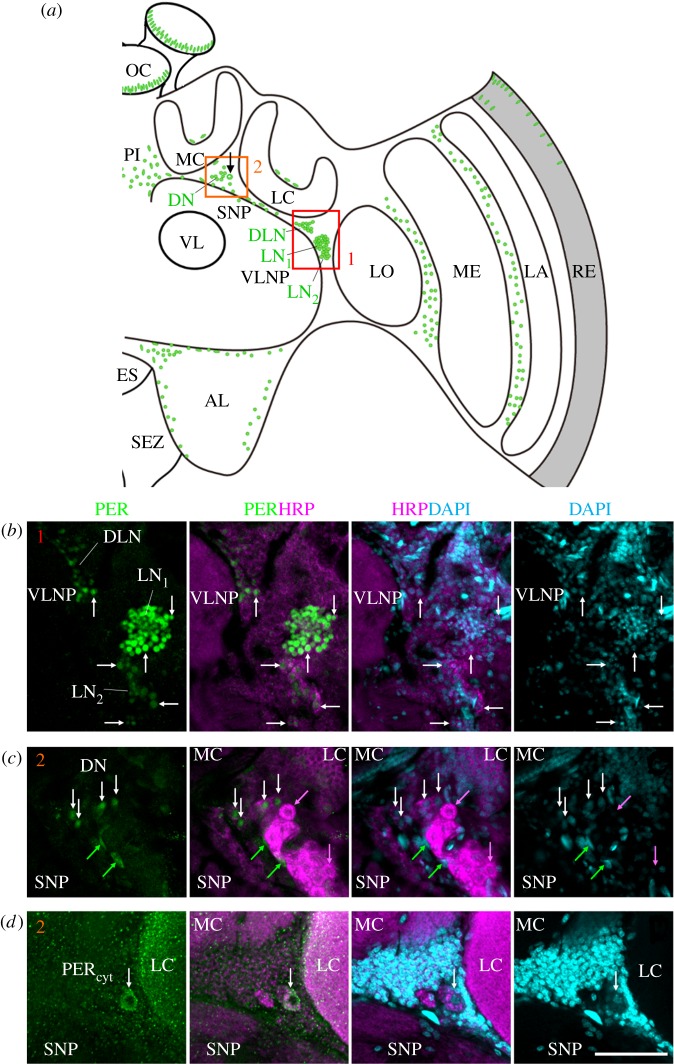
amPER-immunoreactive neurons in the honeybee brain. (*a*) Schematic presentation of amPER-positive cells (green) in the right brain hemisphere. amPER-positive neurons were present in the dorsal and lateral central brain and named DN, DLN, LN_1_ and LN_2_ according to their location and the nomenclature in *Drosophila melanogaster*. The most conspicuous LN_1_ are shown together with the LN_2_ and DLN in (*b*) (red rectangle 1 in (*a*)) and in more detail in figure 4*a*,*b* and electronic supplementary material, figure S3). The DN and one amPER-positive cell with cytoplasmic staining (arrow in (*a*)) are shown in (*c*) and (*d*) (orange rectangle 2 in (*a*)). Note that (*d*) is located slightly posterior of (*c*). (*b*) amPER-positive neurons in the dorsolateral and lateral brain (DLN, LN_1_, LN_2_). White arrows point to selected neurons of all three groups. All PER cells were also marked by HRP and DAPI, although the staining was sometimes weak (overlay of two confocal stacks). (*c*) PER-positive cells in the dorsal brain between medial (MC) and lateral calyces (LC) and the superior neuropils (SNP). White arrows point to four PER-positive neurons, the cytoplasm of which is also labelled by HRP and the nuclei weakly by DAPI. Green arrows point to two PER-positive but HRP-negative glial cells, the nuclei of which are also labelled by DAPI. Magenta arrows point to two HRP-positive neurons that are PER-negative (one single confocal stack). (*d*) A large PER-positive neuron with cytoplasmic staining (PER_cyt_), which is located in the dorsal brain between medial (MC) and lateral calyces (LC) and the superior neuropils (SNP). The cytoplasm of this neuron is co-stained with HRP and its nucleus is weakly DAPI-positive (overlay of two confocal stacks). AL, antennal lobe; ES, esophageal foramen; LA, lamina; LC, lateral calyx; LO, lobula; MC, medial calyx; ME, medulla; OC, ocelli; PI, pars intercerebralis; RE, retina; SEZ, subesophageal zone; SNP, superior neuropils; VL, vertical lobe of the mushroom body; VLNP, ventrolateral neuropils. All photos in (*b*–*d*) were taken from foragers' brain. Scale bars, 30 µm. Pictures are taken with a 10× objective (numerical aperture: 0.3); distance of *z*-stacks 2.5; overlay of three stacks.

**Figure 4.**
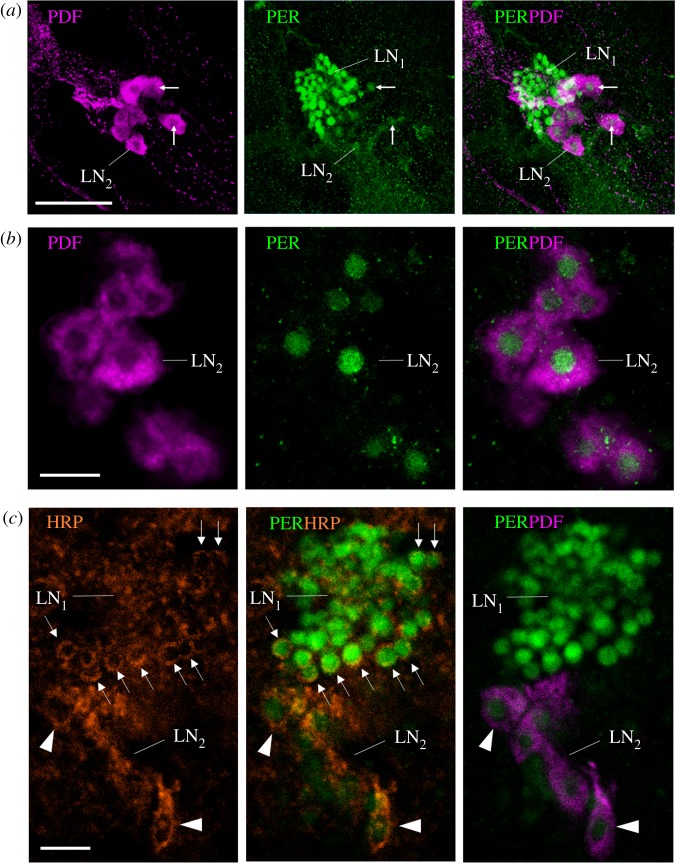
A finer characterization of the LN_2_. (*a*) Lateral neurons LN_1_ and LN_2_ (overlay of 10 confocal stacks from a vibratome section, distance of *z*-stacks 1.0 µm). All LN_2_ are co-stained with both anti-PER and anti-PDF. White arrows point to selected LN_2_. Scale bar, 30 µm. (*b*) Magnification of the PDF-positive LN_2_ (single confocal stack from a wholemount brain; 10× objective; numerical aperture: 0.30). The brain was scanned in *z* steps of 2.5 µm, and the present image is at a depth of 85 µm from the anterior surface. PER is confined to the nuclei of the LN_2_. Scale bar, 10 µm. (*c*) Single confocal stack of a vibratome section (the same as in figure 3*b*) showing the LN_1_ and LN_2_ stained by anti-PER, anti-HRP and anti-PDF (same microscope settings as in (*b*)). HRP and PDF are shown in magenta and indicate the size of the neurons. Although HRP does not stain the cytoplasm membrane uniformly, it shows that the LN_1_ are much smaller than the LN_2_. All photos were taken from foragers' brains. Arrows point to single LN_1_ neurons the cytoplasm of which is clearly labelled by the neuronal marker HRP. Arrowheads point to single LN_2_ neurons the cytoplasm of which is HRP positive and additionally labelled by PDF. Scale bar, 10 µm.

**Figure 5.**
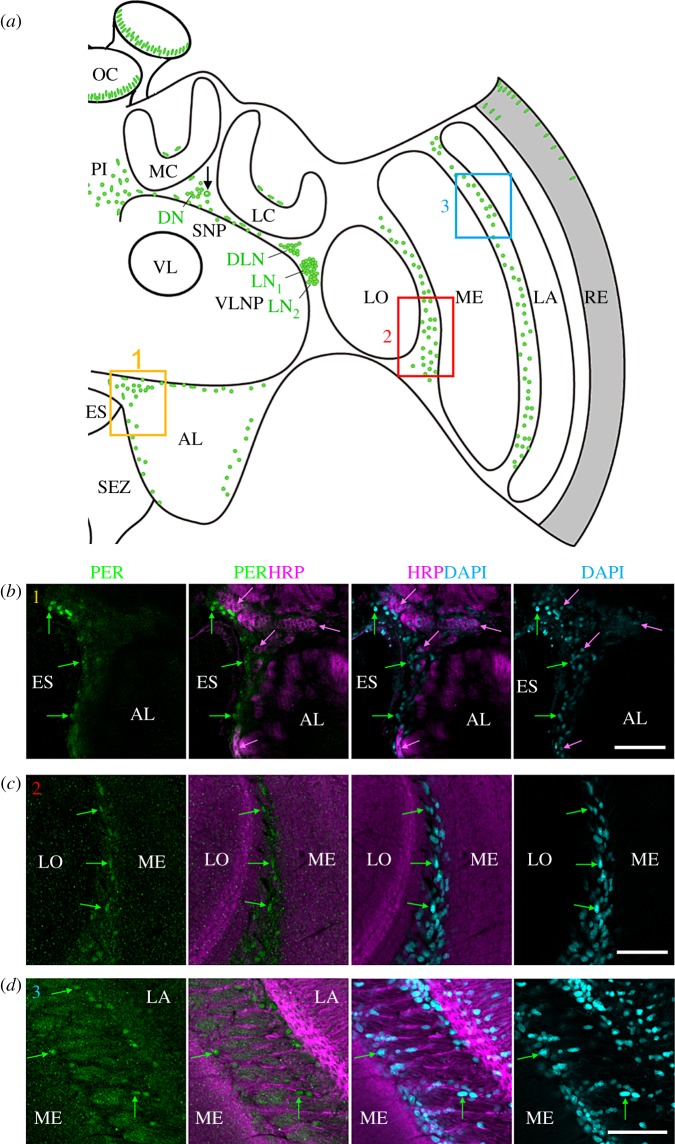
Putative amPER-positive glia cells. (*a*) Schematic presentation of amPER-positive cells (green) in the right brain hemisphere. Putative amPER-positive glial cells were present throughout the cortex of the brain, dorsally of the calyces (MC and LC), at the border of the antennal lobe (AL) (yellow rectangle 1), between lobula (LO) and medulla (ME) (red rectangle 2) and between medulla (ME) and lamina (LA) (blue rectangle 3). (*b*) PER-positive glial cells around the esophageal foramen (ES) close to the antennal lobe (AL) (see inset 1 in (*a*)). Green arrows mark selected PER-positive nuclei that are also stained by DAPI but not by HRP. Selected HRP-positive neurons are marked by magenta arrows. The nuclei of the latter were also stained by DAPI, but clearly less intensively than the glial cells. (*c*) PER-positive glial cells between the lobula (LO) and medulla (ME) (see inset 2 in (*a*)). Labelling as in (*a*). (*d*) PER-positive glial cells between the medulla (ME) and lobula (LO) (see inset 3 in (*a*)). All photos in (*b–d*) were taken from foragers' brains. Please note that only subsets of glia cells are PER positive. Scale bars, 30 µm.

**Figure 6.**
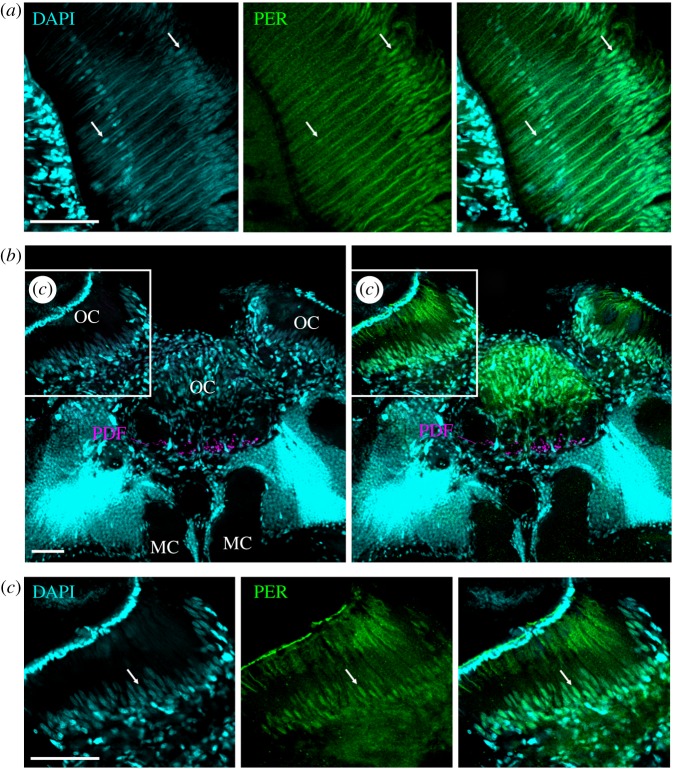
PER-ir staining in photoreceptor cells in the compound eyes and ocelli. (*a*) amPER (green) and DAPI (cyan) double-labelling in the retina of a wholemount brain. DAPI labels the photoreceptor nuclei at a proximal and distal level of the retina (white arrows). At the proximal level only one nuclei per ommatidium is labelled that should correspond to photoreceptor cell 9, whereas in the distal layer 8 nuclei were labelled that are all at slightly different depths in the ommatidium. These should correspond to the nuclei of photoreceptor cells 1–8. The nuclei at the distal level were clearly co-labelled by the amPER antibody, the nuclei of the proximal level only faintly, and in some the PER labelling was barely visible. Note that the cornea of the compound eye is detached from the retina due to the wholemount preparation. (*b*) amPER, DAPI and PDF (magenta) labelling in superior median brain and the ocelli (OC). The strongest DAPI labelling is found in the Kenyon cells of the mushroom bodies, just dorsally of the median calyces (MC). Strong DAPI labelling is also present in many glial cells in the ocelli. PDF extends into the base of all three ocelli. Here, it can only be seen in the median ocellus. (*c*) A higher magnification of the left ocellus as indicated in the inset in (*b*). All photos were taken from foragers' brains. A white arrow points to an arbitrarily chosen nucleus of one photoreceptor cell. Scale bars, 30 µm. Pictures are taken with a 10× objective (numerical aperture: 0.3); distance of *z*-stacks 2.0; overlay of two stacks.

**Figure 7.**
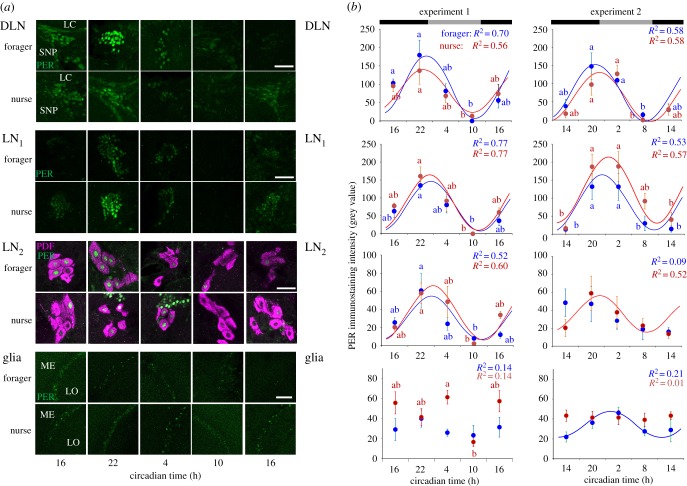
Oscillations in amPER immunostaining intensity in neuronal clusters and glial cells in foragers and nurses sampled around-the-clock. (*a*) Representative microphotos of vibratome-sectioned brains double-stained with anti-amPER (green) and anti-PDF (magenta) antibodies. Cell cluster names are as described in figure 3*a*. Samples were collected every 6 h for a total of five time points. Scale bars, 20 µm. (*b*) Mean immunostaining intensity ± s.e.m., and fitted cosine model curves for Trial 1 (left, *n* = 4 brains/time point) and Trial 2 (right, *n* = 6). Values for nurses are depicted in red, and for foragers in blue. For each curve the calculated colour coded *R*^2^ from regression with a cosine model is indicated in the upper right corner. Statistically significant (*p* < 0.05) cosine models are depicted by a continuous line. Time points with different small letters are significantly different in Kruskal–Wallis and Nemenyi post hoc test (*p* < 0.05). Pictures are taken with a 40× objective; distance of *z*-stacks 1.0.

Immunocytochemistry of 242 vibratome sections and 16 wholemount honeybee brains showed a consistent neuroanatomical pattern of amPER-ir staining (figures 3–7). We detected amPER-ir cells throughout the cell body layer of the central brain (except for the subesophageal ganglion), in the optic lobes, as well as in the compound eyes and ocelli (figure 3*a*). We did not detect obvious differences between the pattern of PER-ir staining in nurses and foragers (figure 7). Immunostaining was mostly nuclear (figures 3–7), with the exception of one to two large cells between the calyces and the alpha/beta lobes of the mushroom bodies (MB) of each brain hemisphere that are stained occasionally (in 12.5% of the wholemount brains and 5% of the vibratome sections; black arrow in figure 3*a*,*d*; all the staining shown in figures 3–5 are from brains of forager bees).

The most conspicuous and intense staining was seen in a large cluster consisting of 105–120 small cells located between the anterior ventrolateral protocerebrum (AVLP) of the ventrolateral neuropils (VLNP) and the optic lobes. A second, smaller cluster of cells was located ventrally, just adjacent, to the large cluster. This second cell group consisted of only 14–16 cells with larger nuclei that were less intensively immunostained with the anti-amPER antibody. According to their position, we call the cells in these clusters ‘lateral neurons’, LN_1_ and LN_2_, respectively (figures 3*a*,*b* and 4). We used amPER immunoreactivity to measure nucleus diameter for LN_1_ and LN_2_ cells in three brains and found it to be smaller for the LN_1_ cells (5.0 ± 1.3 µm (± s.d.), *n* = 45 cell nuclei) compared with the LN_2_ (8.6 ± 1.1 µm, *n* = 38 nuclei; two-way ANOVA, cell type: *F* = 174.5, *p* < 0.0001; brain: *F* = 3.3, *p* = 0.07; cell type × brain: *F* = 3.4, *p* = 0.07).

Another cell cluster consisting of 60–75 cells, which were also less intensively stained, was located dorsally of the LN. This cell cluster was clearly separated from the large LN_1_ and LN_2_ clusters, and the cells appeared less tightly packed and ranged from the anterior to the posterior part of the brain. Only the ones in the anterior part of the brain are drawn in figure 3*a* and shown in the following figures. According to its position in the brain, we call this cluster ‘dorsal lateral neurons', DLN. The amPER-ir nuclei of the cells in clusters LN_1_, LN_2_ and DLN had a round shape.

### 3.4. Some lateral neurons (LN_2_) of *Apis mellifera* co-express the neuropeptide PDF

The two LN clusters in the frontal–lateral honeybee brain are reminiscent of the lateral clock neurons of *D. melanogaster*, and this is one of the reasons for naming them accordingly. In *D. melanogaster*, one group of the LN, the ventrolateral neurons (LN_v_), co-express the neuropeptide pigment-dispersing factor (PDF). PDF is also important in the circadian system of other insects [29,42–45]. Therefore, we asked whether some of the amPER-ir cells also co-express PDF and performed double-labelling with the new anti-amPER antibody and antibodies against PDF. The anti-PDF labelled the ventral group of 14–16 amPER-ir neurons that had larger nuclei (LN_2_, figure 4*a*,*b*). As in *D. melanogaster*, PDF was cytoplasmic and confirmed the HRP results that the ventral group of amPER-ir neurons are large. Nevertheless, like in *D. melanogaster* they are not homogenous in size, but cannot be clearly divided into two groups of large and small cells [21,46]. The smaller LN_1_ cells that show the strongest amPER immunostaining, and are located dorsally of the LN_2_, were not co-stained with anti-PDF. Similarly, the DLN and dorsal neuron (DN) clusters were not immunostained with the anti-PDF antibody.

### 3.5. amPER-immunoreactive neurons in the dorsal brain and in other brain areas

Similarly, round amPER-ir nuclei were also found in the dorsal protocerebrum between the medial and lateral calyces and close to the cells with cytoplasmic staining (named ‘dorsal neurons’ (DN, figure 3*a*). These cells (*n* = approximately 15) were less conspicuous than the LN and DLN clusters and sometimes hard to distinguish from other amPER-ir nuclei that were aligned along a line passing ventrally to calyces of the MB, just above the dorsal protocerebrum (=superior neuropils, SNP) and the vertical lobe (VL) of the MB (figure 3*a*,*c*,*d*). Some of the latter nucleic areas were larger and had a longish shape (figure 3*b–d*). Strong nuclear amPER-ir staining was also seen in various locations in the cell body layer of the antennal lobes (figure 5*a*,*b*), as well as in the optic lobes (figure 5*a*,*c*,*d*). In the optic lobes, amPER-ir cells were concentrated mainly in two areas: between the lobula and medulla (figure 5*c*), and between the medulla and the lamina (figure 5*d*). In all these locations, the nuclei had round or longish shapes (figure 5*b–d*). In some brains, we furthermore found a small number of amPER-ir nuclei with elongated shape above the calyces (figure 3*a*). We further examined the presence of amPER-ir signal in the compound eyes and the ocelli. In two brains, in which the ocelli and large parts of the retina remained attached to the brain, we found that the nuclei of the photoreceptor cells in the compound eyes and ocelli were stained by the amPER antibody (figures 3*a* and 6).

### 3.6. Horseradish peroxidase immunocytochemistry reveals that many of the amPER-ir cells are of glial nature

Next, we tested whether the amPER-ir cells are neurons or glial cells. Staining with an antibody against HRP is suited to distinguish insect neurons from glial cells [47]. The HRP antibody recognizes a carbohydrate residue of the neuron-specific cell surface protein Nervana [48]. Therefore, HRP labels the surface of all neurons (cell bodies and neurites) but not glial cells and leaves the nuclei unlabelled. We performed the HRP staining on vibratome sections of two of the amPER-immunostained brains. To visualize the nuclei of neurons and glial cells, we counterstained with DAPI, which binds to DNA [49]. We found that the cell bodies of the majority of the amPER-ir cells were not HRP positive, which classified them as putative glial cells (figures 3–5). This was the case for the amPER-ir cells in the antennal lobes (figure 5*b*), the cells in the optic lobes (figure 5*c*,*d*) and many cells aligned in a row between the dorsal protocerebrum and the MB calyces (figure 3*a*), the cells in the *pars intercerebralis* (electronic supplementary material, figure S2) and the cells above the calyces (not shown).

Among the amPER-ir positive cells that were also HRP positive (i.e. neuronal cells) were the weakly amPER-ir cells between the medial and lateral calyces (DN, figure 3*b*), among which were also the occasional one to two cells with cytoplasmic amPER-ir staining (figure 3*d*). Importantly, the LN_1_, LN_2_ and DLN were also HRP positive (figure 3*b*). For better clarity, all amPER-ir neuronal clusters are labelled with green letters in figure 3*a*. HRP staining showed that the LN_2_ had not only larger nuclei but also rather large cell bodies (figure 4*c*, electronic supplementary material, figure S3). The cytoplasmic area of the DLN and LN_1_ was considerably smaller, making HRP difficult to detect. This was especially true for the tightly packed LN_1_ cluster. Nevertheless, we could unequivocally reveal HRP staining in the cells located at the border of the cluster (marked by arrows in figure 3*b*, see also figure 4*c* and electronic supplementary material, figure S3). Thus, we conclude that the LN_1_ cells are most likely of neuronal origin; we cannot exclude that single glial cells are among them. Furthermore, we see putative glial cells in close vicinity to the LN_1_ and also the LN_2_ (figure 3*b*, electronic supplementary material, figure S3). Processes from these glial cells may intermingle with the processes of the LN clock neurons. The DAPI staining confirmed that amPER-ir signal was always confined to the nucleus (except for the one to two neurons between the calyces). This was true for amPER-ir glial cells and neurons. Interestingly, DAPI staining tended to be stronger in amPER-ir glial cells than in neurons (see figures 3–5; electronic supplementary material, figure S3).

### 3.7. amPER-ir intensity in neuronal clusters shows similar oscillations over the day in nurses and foragers

After characterizing the neuroanatomy of amPER immunoreactivity in the honeybee brain, we turned to examine the temporal regulation by amPER immunostaining in nurse and forager bees collected around the day. We first performed a preliminary experiment in which we immunostained only forager brains, and then we performed two trials (each with bees from a different source colony that are therefore expected to differ genetically) of the main experiment in which we immunostained both foragers and nurses. In the preliminary experiment, we measured signal intensity in the LN and DLN clusters. Immunostaining intensity in these clusters varied over the course of the day (Kruskal–Wallis test *χ*^2^ = 16.4 and 10.6, *p* = 0.003 and 0.03, for LN and DLN, respectively; *n* = 4 brains/time point), and with a very similar pattern (electronic supplementary material, figure S4). The strongest amPER-ir signal was measured 9 h after the switch from light to dark (CT 21), and the trough occurred 12 h later at CT 9. After the trough, signal intensity increased again consistent with a model in which amPER immunoreactivity in these two clusters oscillates with a period of about a day. In the two trials of the main experiment, we added measurements of amPER-ir signal intensity of glial cells between the lobula and medulla. The patterns of amPER-ir in the LN and DLN of foragers were very similar to that recorded in the preliminary experiment. amPER-ir signal intensity varied over the day with a peak towards the end of the subjective night (CT 22) and a trough 12 h later at the end of the subjective day (CT 10) (figure 7). The phase of amPER oscillation was similar in all three clusters, but immunostaining intensity was weaker in the PDF-positive LN_2_ (compare the scale in figure 7).

Overall amPER-ir signal was similar in foragers and nurses (two-way ANOVA; task effect: *F* < 2.7, *p* > 0.1, in both trials). Only in the second trial, the amPER-ir levels in the LN_1_ turned out to be slightly lower in foragers than in nurses (figure 7*b*, right panel, *F* = 6.7, *p* = 0.013). In all the analyses, the effect of time of day was significant (two-way ANOVA; time of day effect: *F* > 3.0, *p* < 0.03), and there was no interaction between task and time of day (*F* < 1.65, *p* > 0.19 for all analyses) indicating that the temporal pattern is similar in nurses and foragers for all tested clusters. In both nurses and foragers, cosine models with a period of 24 h explained a significant proportion of the variation in all three amPER-ir neuronal clusters (*R^2^* = 0.52–0.77, with the exception of LN_2_ of foragers in the second trial; figure 7*b*). The cosine analyses are consistent with a period of about 24 h for both nurses and foragers. Although the average phase is similar, it is possible that the PER-ir oscillation is less synchronized among individual nurse bees compared to foragers. If true, we would expect to see greater variability in staining intensity for a given cluster in nurses compared with foragers. To estimate variability among PER-ir cells within a cluster, we compared the coefficient of variation (CV) of amPER-ir intensity for the LN_2_ (for which the PDF immunostaining allowed us to find and measure all cells, even when amPER-ir signal is very low or absent, as needed for meaningful CV analyses; see Material and methods) across the five time points for both nurses and foragers. We found that the CV varied with time, but did not differ between foragers and nurses in both trials (two-way ANOVA; task: *F* = 0.0003 and 0.24, *p* = 0.99 and 0.63; time of day: *F* = 7.42 and 3.59, *p* = 0.0003 and 0.012; interaction between task and time: *F* = 0.7 and 1.17, *p* = 0.59 and 0.33; first and second trial, respectively; data not shown). These analyses suggest that the synchronization among PER-ir cells within each time point is similar for nurses and foragers. Taken together, the three experiments (including the preliminary experiment) revealed robust amPER-ir oscillations in the LN and DLN clusters of both nurses and foragers. The intensity of PER immunoreactivity, the phase of cycling and the cell synchronization appeared similar for foragers that show strong activity rhythms and for nurses that are active around-the-clock.

We also measured amPER-ir intensity in one group of glial cells located between the medulla and lobula of the optic lobes (figure 7*b*, lower panel). In both trials, overall amPER-ir intensity was higher in nurses compared to foragers (two-way ANOVA; task: *F* = 11.7, *p* = 0.0018, *F* = 6.27, *p* = 0.016, for the first and second trial, respectively) and there seemed to be a weak circadian pattern in amPER-ir intensity in foragers but not nurses (two-way ANOVA; time: *F* = 3.6 and 1.13, *p* = 0.016 and 0.35; time × task: *F* = 2.8 and 1.33, *p* = 0.043 and 0.27, for the first and second trial, respectively). The peak in the foragers occurred in the late subjective night (first trial) or early subjective day (second trial) and the apparent peak and trough were 12 h apart. Nevertheless, there was no significant time effect in a separate Kruskal–Wallis test for only the foragers in the two trials (*χ*^2^ = 2.8 and 7.0, *p* = 0.6 and 0.13, respectively). amPER-ir intensity varied over time in nurses in the first trial (Kruskal–Wallis test, *χ*^2^ = 10.8, *p* = 0.03), but the pattern was not circadian, and in the second trial amPER-ir levels appeared similar throughout the day. In sum, there was no significant circadian cycling in amPER immunoreactivity in these glial cells. Nevertheless, a pattern suggesting oscillation with a period of about a day was present in foragers but absent in nurses.

## 4. Discussion

Immunostaining the honeybee brain with a new and specific anti-amPER antibody has led to four important discoveries. First, we have provided the best available description of the circadian network in the honeybee brain, setting the stage for neuroanatomical studies on clock regulated complex behaviours such as time memory, time-compensated sun-compass navigation and dance communication, for which the honeybee provides an outstanding model system. Additional details on the neuroanatomy of the clock network will be reported in Beer *et al*. [46]. Second, we have revealed significant neuroanatomical similarities between PER immunoreactivity in the honeybee, *Drosophila melanogaster* and other insects, which suggest that there are common anatomical organization principles in the insect clock that have not been appreciated before. Third, in contrast to *Drosophila* but similar to mammals, amPER was detected almost exclusively in the nucleus at all times of day. This observation is consistent with the premise that amPER entry into the nucleus is not gated in the honeybee. This finding adds to earlier evidence that in some important ways the honeybee clock is more similar to that of mammals than to *Drosophila*. The emerging species-specific variability in the circadian clockwork raises the intriguing hypothesis that having different clock genes has an adaptive function. Fourth, the intensity of amPER-ir signal in three core clusters of lateral neurons shows similar strong oscillations with peak levels during the late subjective night in nurses and foragers. This finding lends credence to the hypothesis that circadian regulation with an appropriate phase is beneficial, and perhaps crucial, even in animals that are active around-the-clock in a constant physical environment.

Our rigorous validation of the new anti-amPER antibody (combining WB, IP with anti-amPer and anti-amCry antibodies, immunocytochemistry on wild-type and *per^01^* mutant *Drosophila* flies and on honeybee brains after pre-absorbing the antibody with tissue of *Drosophila per^01^* mutants) shows that the antibody specifically recognizes the PER protein. Peak abundances in both whole brain WB and immunocytochemistry occurred late in the night (figures 2 and 7, electronic supplementary material, figure S4), suggesting a delay between the phases of amPer mRNA and protein abundance (for the mRNA phase see, for example, [16,24,39,40,50]).

Our immunostainings confirm and significantly extend earlier studies in honeybees that applied antisera raised against PER from other insect species, specifically the full-length *D. melanogaster* PER protein or a synthetic 14-mer peptide corresponding to a fragment of the most conserved region of the *Antheraea pernyi* PER [21,29]. AmPER has 55% amino acid sequence similarity to *Drosophila* PER [39] and differs in only one residue from the corresponding 14-mer peptide of *A. pernyi* making it very likely that these antisera recognize amPER, at least in some configurations. The successful immunostaining of the well-characterized PER-expressing neurons in *Drosophila* brains incubated with the new amPER antibody further confirms the similarity of the bee and fly PER proteins. Similar to the immunostaining with the anti-dmPER [21], we found many cells with nuclear PER-ir in the optic lobes and central brain, but in our earlier study we were not confident concerning the specificity of PER-ir in these parts of the brain. Indeed, cross-species antibodies may provide weaker epitope recognition, could fail to immunostain some PER-expressing cells and increase the risk of false positives. For example, it was recently shown that antisera generated against part of the *D. melanogaster* PER successfully immunostained clock neurons in related *Drosophila* species such as *D. simulans* and *D. yakuba*, but not in the less taxonomically related *D. ananassae*, *D. triauraria*, *D. pseudoobscura*, *D. willistoni*, *D. virilis*, *D. littoralis* and *D. ezoana* [51]. Similarly, immunostaining of wild-type *D. melanogaster* brains with the anti-amPER antibody was rather weak even after incubation for a period of 3 days. The lower specificity of the anti-*Drosophila* and *A. pernyi* PER antibodies [21,29] may compromise detection in cells showing weak nuclear amPER labelling such as the LN_2_ cells that co-express PDF.

### 4.1. amPER immunoreactivity reveals common neuroanatomical principles in the insect circadian clock

The pattern of amPER immunoreactivity in the honeybee suggests considerable similarity to the pattern of dmPER immunoreactivity in *D. melanogaster* (reviewed by [38,52]). In both species, strong PER-ir signal is present in lateral and dorsal neurons, in many putative glial cells throughout the cortex of the brain, the antennal lobes and the optic lobes, and in photoreceptor cells of the compound eyes and the ocelli. A more detailed examination revealed additional similarities. The lateral central brain of *Drosophila* includes three distinct neuronal clusters, the ventrally located small and large LN_v_ and the more dorsal LN_d_. Additionally, three clusters of dmPER-ir neurons are located in the dorsal brain, named dorsal neurons DN_1_, DN_2_ and DN_3_. The ventrally located small and large LN_v_ of *Drosophila* co-express the neuropeptide PDF (except for one small LN_v_ that is PDF-negative). Similarly, the ventral group of lateral neurons in *A. mellifera* that we named LN_2_ co-express PDF and are most probably homologous to the LN_v_ of *D. melanogaster*. Like the LN_v_ of *D. melanogaster*, the LN_2_ neurons of *A. mellifera* are of different sizes, although we could not classify them into small and large neurons since they contain also medium-sized cells [46]. Ablation and transplantation studies in the cockroach located its circadian clock in the accessory medulla (AMe) ventrally between the lobula and medulla of the optic lobes [44,53]. The AMe is densely innervated by PDF-ir neurons in all insects investigated so far [54,55]. Furthermore, in flies, manipulation of the PDF-positive lateral neurons strongly affects circadian rhythmicity [38,56]. Although similar studies have not been performed for honeybees, injection of PDF or even just saline into the vicinity of the PDF-ir cells affected circadian rhythms, suggesting that this brain area is important for circadian rhythmicity [46]. The similar oscillations in bees from three different colonies (figure 7, electronic supplementary material, figure S4), that differ genetically, provide additional support for the premise that these clusters of lateral neurons are key components of the central circadian network of the honeybee. Taken together, these observations mark the area between the optic lobes and central brain as a pivotal component of the honeybee circadian clock.

Although the general organization is similar between the honeybee and *Drosophila*, the honeybee clock network contains significantly more cells. The number of PER/PDF co-expressing neurons is eight per hemisphere in *D. melanogaster* and about twice this number in the honeybee. The difference is even larger for the PDF-negative PER-ir lateral neurons: *D. melanogaster* possesses six LN_d_ neurons whereas the similarly located LN_1_ cluster in the honeybee consists of 105–120 neurons. Owing to this large difference in number, we cannot be certain whether the fly LN_d_ and bee LN_1_ are homologous in nature and decided to give them different names. The more dorsally located DLN of *A. mellifera* includes approximately 70 cells that are reminiscent of the approximately 40 DN_3_ cells of *D. melanogaster*, because they are more spread throughout the dorsolateral brain than the two lateral cell clusters and extend into more posterior parts of the brain. Thus, they are sitting like a saddle on top of the lateral protocerebrum very similar to the DN_3_ of *D. melanogaster*. The bee DLN cells seem to be located less dorsally than the fly DN_3_. This may be explained by the much larger bee calyces that dominate the dorsal brain (figure 3*a*) and may cause these DLN cells to appear lower and more lateral than in *Drosophila*. In the case of the approximately 15 bee DN cells, we are more confident that these correspond to the fly DN_1_. These cells are not only similar in number, but are also located in the same position anterior of the calyces and dorsal of the PDF fibres—not touching the latter. So far, we have not been able to unequivocally identify neurons in the bee dorsal brain that may correspond to the two fly DN_2_, which virtually sit on the PDF fibres. This does not mean that they do not exist.

The similarities between the circadian network of *Drosophila* (Diptera) and *Apis* (Hymenoptera) prompted us to compare our findings to PER immunoreactivity in species from additional insect orders. We focused on studies using validated antibodies that were raised against a protein or peptide sequence specific to the studied species because immunostaining is more likely to represent genuine PER expression (see above). A very similar pattern with prominent PER-ir signal in clusters of dorsal and lateral neurons, and PDF expressing lateral neurons sending fibres to both the optic lobes and the dorsal brain is also found in diverse species such as the German cockroach *Blattella germanica* (Blattodea [57]) and the blow fly *Protophormia terraenovae* (Diptera [56]). A more variable pattern is seen in species from the Lepidoptera. In the hawkmoth *Manduca sexta*, PER expression was described with an antibody directed towards a 358 amino acid specific msPER peptide containing the PAS-domain. Immunostaining with this antibody and complementary *msPer in situ* hybridizations revealed a pattern very similar to our findings for the honeybee. The anti-msPER antibody immunostained the nuclei of a conspicuous cell cluster in the lateral brain that consisted of 100–200 small neurons that is reminiscent of the LN_1_ cluster of the honeybee brain. Furthermore, the nuclei of the photoreceptor cells in the compound eyes, and round or longish nuclei of many glial cells throughout the brain, the antennal and optic lobes, were stained by both immunocytochemistry and *in situ* hybridization. An additional similarity between the hawkmoth and the honeybee is cytoplasmic staining in one to two large (leu-enkephalin- and corazonin-positive neurosecretory) cells in the *pars lateralis* of the dorsal brain [58]. These neurosecretory cells were less intensively stained than the other cells with nuclear PER and did not show daily cycling in PER abundance in their terminals in the corpora cardiaca. The size, location and cytoplasmic staining of these cells are similar to amPER-ir neurons that we found in the current study and the ‘DPER-L’ and APER-L neurons described previously [21,29]. Moreover, cells with a similar size and location were also immunostained with antibodies directed against the *Drosophila* CYCLE protein (Bloch and Ben David 2008, unpublished results). Cytoplasmic PER-ir signal in large cells in the *pars lateralis* has also been described in insect species from diverse orders; in some cases, this cytoplasmic PER-ir signal cycles over the day [29,57,59–61]. However, immunostaining in these large cells needs to be interpreted with caution because their location and morphology suggest that they are neurosecretory cells of the *pars lateralis*, the cytoplasm of which is rich in peptides and proteins that might weakly cross-react with anti-PER antibodies and be regulated in a circadian manner.

The only notable difference between PER-ir signal in the hawkmoth and the honeybee brain is that the PER and PDF-positive LN_2_ cell cluster is absent in the hawkmoth. An overall similar pattern with many PER-ir cells in the dorsal and lateral brain, as well as in the optic lobes, but with cytoplasmic PER-ir signal in large cells in the *pars intercerebralis* rather than in the *pars lateralis*, was recently reported for the Mediterranean flour moth, *Ephestia kuehniella* [62]. It is notable however that a different pattern was reported for *Bombyx mori*, *A. pernyi* and *Danaus plexippus*. In these phylogenetically derived lepidopterans PER-ir signal is limited to the cytoplasm of a small number of cells in the central brain, and to the nucleus of photoreceptor cells, with no immunostaining in dorsal and lateral neurons. Given that *E. kuehniella* is phylogenetically a basal Lepidoptera species, it was hypothesized that the ancestral lepidopteran clock possessed clock neurons in the lateral and dorsal brain but the number of PER-expressing cells was reduced with clock neurons disappearing from certain brain regions [62]. Another possibility is that the anti-*A. pernyi* antibody that was used in these studies, and which was raised against a short peptide, does not recognize certain configurations of PER, including in the nuclei of lateral and dorsal neurons. Indeed, although the 14 amino acids PER peptide of *A. pernyi* differs in only a single amino acid from the corresponding sequence of the honeybee, this antibody failed to immunostain the lateral neurons, and glia all over the brain of the honeybee [21,29]. Nevertheless, the clock of all the lepidopterans differs from that of other insects in the lack of lateral neurons expressing both PER and PDF [59,63], suggesting that some modifications in the circadian clock system occurred early in the evolution of this lineage. PER-ir staining has been characterized in many other species representing diverse insect orders. However, given that these studies typically used cross-species antibodies (most commonly those raised against the *D. melanogaster* or *A. pernyi* PER proteins) the interpretation of the immunostaining is more difficult.

The neuroanatomy revealed here for the honeybee circadian clock shows striking similarities to that of fruit flies, cockroaches, beetles and hawkmoths. Given that these species represent diverse orders, we suggest that there is an ancient and common neuroanatomical organization for the insect clock network. Central pacemakers are located in clusters of lateral and dorsal neurons and in the optic lobes, and PDF that is expressed in a subset of the lateral neurons serves as a major coupling signal. During evolution this ancestral network structure could have been modified, as suggested for example in the Lepidoptera.

### 4.2. Brain amPER immunoreactivity cycles over the day but does not show gated translocation from the cytoplasm to the nucleus

We observed significant oscillations in amPER abundance both on the whole brain level (WB, figure 2) and in the lateral and dorsal neurons of the honeybee (figure 7 and electronic supplementary material, figure S4). The phase of cycling is consistent with that reported by Bloch *et al*. [21] and with the phase expected based on the peak of *amPer* transcript abundance, which typically occurs around mid- to late night (reviewed in [8,12]). The strong oscillations in the neuronal clusters in bees from three different source colonies (which varied genetically) provide additional evidence that these cell clusters are pivotal components of the circadian network of the honeybee. Interestingly, amPER-ir signal was not detected in the cytoplasm. We cannot unequivocally exclude the possibility that our time resolution (sampling every six hours) or spatial resolution were not sufficient to detect cytoplasmic amPER accumulation in these cells. However, similar sampling did detect dmPER in the cytoplasm of clock neurons in *Drosophila* [64]. These analyses together with sporadic immunostaining of brains collected at additional time points suggest that in contrast to *Drosophila*, in the honeybees there is no or very little accumulation of PER in the cytoplasm prior to its translocation into the nucleus. This notion is consistent with a similar lack of cytoplasmic accumulation recently reported for the mammalian PER orthologue mPER2. Smyllie *et al*. [65] generated a knock-in mouse expressing a PER2::VENUS *in vivo* reporter that did not compromise the functioning of PER2 in cells of the mammalian suprachiasmatic nucleus (SCN). PER2::VENUS abundance in these mice varied rhythmically in the nuclei of SCN neurons, but was virtually absent from the cytoplasm at all time points. This apparent similarity of the bee with the mouse rather than with *Drosophila* adds to earlier evidence that its molecular clockwork is in many ways more similar to mammals than to *Drosophila* [6]. As in mammals, the honeybee genome does not encode orthologues to *Tim1* and *Cry-d* (insect *Cry1*), but rather encodes a mammalian-like CRY (*Cry-m*/*insect Cry2*), that our IP (figure 1) confirms to interact with PER and repress the transcriptional activators CYC and CLK [6,9,66]. Smyllie *et al.* [65] explained the contrasting behaviour for the fly and mouse PER by the proteins having different hetero-dimerization partners (dTim1 and mammalian-type CRY, respectively). Translocation from the cytoplasm to the nucleus was also not detected in most other insect species in which PER immunoreactivity was studied, in many of which PER-ir signal was always in the cytoplasm [29,57–60,67–69]. This raises the interesting and testable hypothesis that the variation in cytoplasmic or nuclear location of PER immunoreactivity may be at least partially explained by the variability in PER dimerization partners seen in different species (Tim1, Cry-m or both).

### 4.3. Around-the-clock active nurses show strong oscillations in amPER protein abundance in the lateral neurons

As suggested above, coupled clusters of lateral and dorsal neurons compose the circadian network in the honeybee as in other insects. We found that the three major clusters in terms of cell number and PER-ir intensity (LN_1_, LN_2_ and DLN) show similarly strong oscillations of amPER immunoreactivity in nurses and foragers. These results suggest that the core circadian network functions similarly in bees with and without overt circadian rhythms in locomotor activity and explains earlier evidence suggesting that nurses in the hive can measure time. The first such evidence is that nurses with attenuated or no circadian rhythms in the hive switch to activity with strong circadian rhythms shortly after isolation in a constant laboratory environment. These rhythms are in phase with the ambient day–night cycle irrespective of the time they are transferred, suggesting that they have functional entrainable clocks ([15,16]; see Figure 1.3.2 in [8]). In addition, about 160 brain transcripts oscillate with a circadian rhythm in nurses collected from a constant laboratory environment, suggesting that their expression or stability is circadianly regulated [17]. Thus, plasticity in physiological and behavioural outputs such as locomotor activity is achieved not by shutting down the central clock network but rather by flexibility in the way the circadian network is coupled to other clock cells and to output pathways.

Given that nurses are active with weak or no circadian rhythms in a constantly dark and tightly thermoregulated environment, our findings suggest that circadian organization of at least some processes is crucial even under such seemingly constant conditions. Consistent with this premise are studies with humans and other animals showing that genetic or environmental disturbances to normal circadian rhythms compromise health, metabolism and performance [2,70,71]. It is also possible that a robust internal clock supports plasticity in task performance because it allows nurses to switch more rapidly to tasks such as nectar receiving, guarding or foraging that require circadian rhythms. Thus, keeping an entrained circadian clock may be part of the toolkit allowing bees incredible social plasticity that is crucial for efficient colony performance and improved fitness [72].

Our findings are not easily reconciled with earlier hypotheses proposed to explain the mechanism underlying plasticity in circadian rhythms. These hypotheses include arrest of the nurse clock, masking of clock output by stronger social influences on overt behaviour, uncoupling the internal clock from output pathways and desynchronization among pacemakers composing the circadian network (reviewed in [8,10]). Our study clearly shows that the clock does not stop in around-the-clock active nurse bees (figures 2 and 7). The hypotheses of masking and uncoupling are not easily reconciled with earlier studies showing that whole brain clock gene mRNA abundance cycles in foragers but not in nurses, because at least in their simple form, these hypotheses assume a similar clockwork functioning in rhythmic and arrhythmic individuals. Our findings are also not consistent with desynchronization among pacemakers in the network of nurses (according to this hypothesis clock neurons oscillate with variable phases in nurses, but are ‘in sync’ in foragers) because the major clock neurons show similar synchronization in nurses and foragers. However, oscillations in additional neuron and glial cells need to be characterized, and a better understanding of the contribution of the various amPER-ir cells is needed in order to determine if pacemaker cells are similarly coupled in nurses and foragers.

Our study provides detailed characterization of the honeybee brain clock network and by that sets the stage for future studies on the interactions between the circadian clock network and complex behaviours. Our results also reveal common neuroanatomical organization principles in insects. Similar analyses with specific antibodies for additional species are needed to better understand the conservation and evolutionary modifications in the clock network of various insect lineages. Finally, our findings raise the question as to why animals that are active around-the-clock with weak or no circadian rhythms in a physically constant environment nevertheless need to invest in keeping their internal clock correctly measuring time and being in phase with external day–night cycles.

## Supplementary Material

Supplementary Material

## Data accessibility

DNA sequence of amPER cDNA underlying antibody production: GenBank accession no. KX169182.
