# Supplementary material for: Neuronal circadian clock protein oscillations are similar in behaviourally rhythmic forager honeybees and in arrhythmic nurses
Source: Open Biol. 2017 Jun 14;7(6):170047. doi: 10.1098/rsob.170047 (PMC5493776; doi:10.1098/rsob.170047)
Supplement: Supplementary Material [file rsob170047supp1.pdf]

Supplementary Material

Fuchikawa, T., Beer, K., Linke-Winnebeck, C., Ben-David, R., Kotowoy A' Tsang, V. W. K., Warman, G.R., Winnebeck, E.C., Helfrich-Förster, C., Bloch, G. (2017) Neuronal circadian clock protein oscillations are similar in behaviourally rhythmic forager honey bees and in arrhythmic nurses. *Open Biology* 7: 170047. DOI 10.1098/rsob.170047

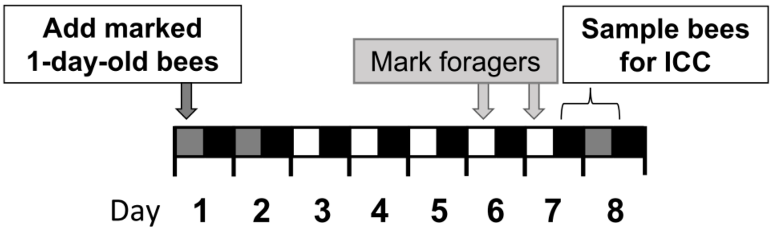

Figure S1. Outline for experiments comparing PER immunoreactivity throughout the day for nurses and foragers. On Day-1 we established a triple-cohort colony, introduced the queen, and paint marked newly emerged workers. The numbers at the bottom of the figure represent the days of the experiment. The bar depicts the illumination regime: black filled box = dark during the night; gray filled box = dark during the day; unfilled box = light on. See text to the specific illumination regime of each trial. The hive entrance was opened at the time of lights-on, and closed at the time of lights-off.

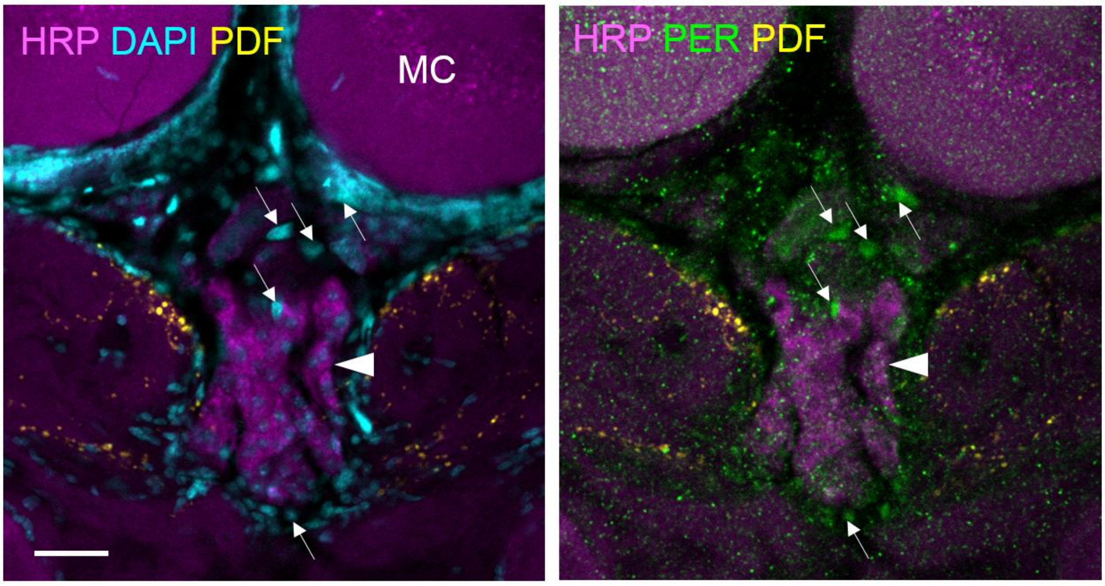

Figure S2. HRP, PER, DAPI and PDF labeling in the *pars intercerebralis*. Overlay of 2 confocal stacks gained from a 150 µm vibratome slice scanned with z steps of 2.5 µm (10x objective with numerical aperture of 0.3). Arrows point to single PER-positive cells that are strongly labelled by DAPI but not by the neuronal marker HRP suggesting that they are glial cells. The arrow points to the large neurosecretory cells in the *pars intercerebralis* which are clearly labeled by HRP. MC medial calyx. Scale bar: 10 µm.

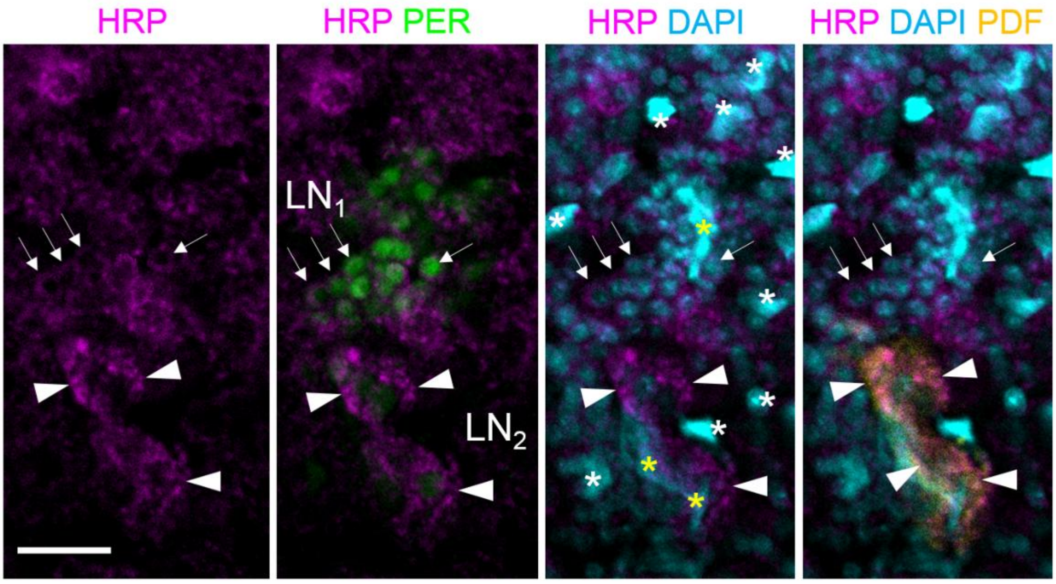

Figure S3: HRP, PER, DAPI and PDF labeling of a single confocal stack in the region of the Lateral Neurons, LN_1 and LN_2. The confocal pictures are gained from a 150 µm vibratome slice scanned with z steps of 2.5 µm (10x objective with numerical aperture of 0.3) at a depth of 27.5 µm from the anterior surface, Arrows point to single LN_1s the cytoplasm of which is clearly labelled by the neuronal marker HRP. Arrowheads point to single LN_2 neurons, the cytoplasm of which is HRP positive and additionally labelled by PDF. The HRP staining is sometimes punctual becuase it does not label the entire cytoplasm membrane of the neurons. Nevertheless, HRP is present in the entire area of the LN_1 and LN_2 cluster that is consistent with the premise that both clusters are of neuronal origin. HRP is absent in several regions in which we find nuclei of irregular shape that are strongly stained by DAPI (asterisks). These are most probably nuclei of glial cells. Some of these cells are close to the Lateral Neurons (yellow asterisks). Scale bar: 10 µm.

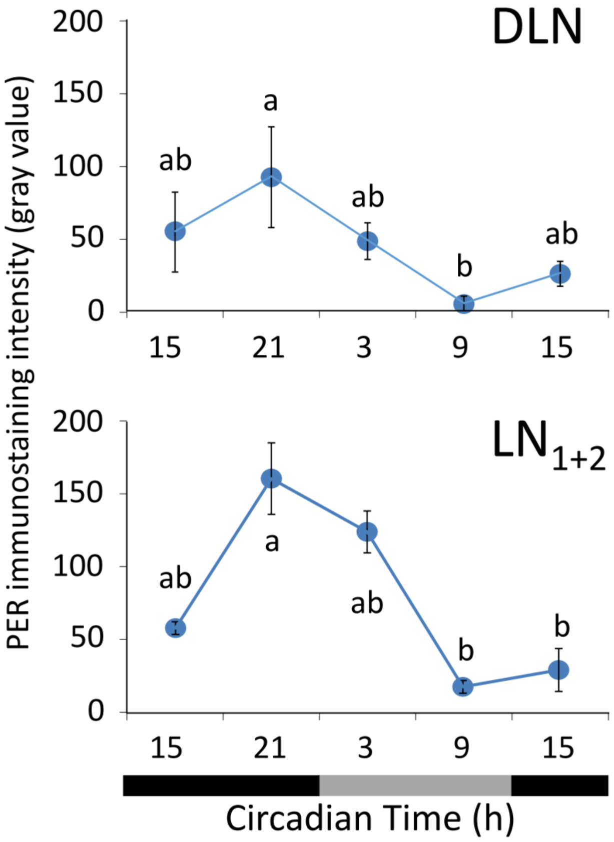

Figure S4. PER-ir immunostaining intensity in foragers collected over the day. Results from this preliminary experiment are shown for the Dorsal Lateral Neurons (DLN) (upper panel, see location in Fig. 3*a*) and the Lateral Neurons 1 and 2 (LN_1 and LN_2, lower panel; given that in this experiment the LN_2 were not double-stained with an anti-PDF antibody, we could not unequivocally distinguish between LN_1 and LN_2 cells. However, given that we measured cells with strong immunostaining intensity, we assume that most, if not all, are LN_1). Sample size was 4 brains/ time point. Error bars indicate SEM. Photoperiod prior to DD is indicated in black and gray bars below the graph. Time points with different small letters are significantly different in Kruskal-Wallis ($\chi 2$ = 10.6, p = 0.03 for DLN, $\chi 2$ = 16.4, p = 0.003 for LN) and Nemenyi post hoc test (p < 0.05).
